# Ovulation induction drug and ovarian cancer: an updated systematic review and meta-analysis

**DOI:** 10.1186/s13048-022-01084-z

**Published:** 2023-01-24

**Authors:** Liang Yu, Jiafan Sun, Qiqin Wang, Wennian Yu, Anqi Wang, Shu Zhu, Wei Xu, Xiuli Wang

**Affiliations:** 1 The First Affiliated Hospital of Nanjing Medical University, The First Clinical Medical College of Nanjing Medical University, Nanjing, 210029 China; 2 Department of Gynecology, Jiangsu Province Hospital, The First Affiliated Hospital of Nanjing Medical University，The first clinical medical college of Nanjing Medical University, Nanjing, 210029 China; 3 Department of Gynecology, Jiangsu Province Hospital, the First Affiliated Hospital of Nanjing Medical University, Nanjing, 210036 China

**Keywords:** Clomiphene, Gonadotrophin, Gonadotropin-releasing hormone analogues, Ovulation induction, Ovarian stimulation, Borderline ovarian tumor, Invasive ovarian cancer, Meta-analysis

## Abstract

**Objective:**

To explore the association between ovulation induction drugs and ovarian cancer.

**Design:**

Systematic review and meta-analysis.

**Setting:**

Not applicable.

**Patient(s):**

Women without ovarian cancer who ever or never underwent ovarian induction.

**Intervention(s):**

An extensive electronic search of the following databases was performed: PubMed, EMBASE, MEDLINE, Google Scholar, Cochrane Library and CNKI, from inception until January 2022. A total of 34 studies fulfilled our inclusion criteria and were included in the final meta-analysis. The odds ratio (OR) and random-effects model were used to estimate the pooled effects. The Newcastle-Ottawa Scale was used to assess the quality of included studies. Funnel plots and Egger tests were used to assess publication bias.

**Main outcomes:**

New diagnosed borderline ovarian tumor (BOT) and invasive ovarian cancer (IOC) between ovulation induction (OI) group and control (CT) group considering fertility outcome, OI cycles and specific OI drugs.

**Results:**

Primarily, there was no significant difference in the incidence of IOC and BOT between the OI and CT groups. Secondly, OI treatment did not increase the risk of IOC and BOT in the multiparous women, nor did it increase the risk of IOC in the nulliparous women. However, the risk of BOT appeared to be higher in nulliparous women treated with OI treatment. Thirdly, among women exposed to OI, the risk of IOC and BOT was higher in nulliparous women than in multiparous women. Fourthly, the risk of IOC did not increase with increasing OI cycles. Lastly, exposure to specific OI drugs also did not contribute to the risk of IOC and BOT.

**Conclusion:**

Overall, OI treatment did not increase the risk of IOC and BOT in most women, regardless of OI drug type and OI cycle. However, nulliparous women treated with OI showed a higher risk of ovarian cancer, necessitating their rigorous monitoring and ongoing follow-up.

**Supplementary Information:**

The online version contains supplementary material available at 10.1186/s13048-022-01084-z.

## Introduction

Infertility affects more than 48.5 million couples worldwide [1–3]. It is emerging as a public health problem, driving the demand for assisted reproductive treatment [4]. Ovulation induction (OI) is a process in which the ovaries are drugged to stimulate the production of many follicles containing eggs, which usually begins early in the menstrual cycle. OI treatment is highly desirable, especially for isolated anovulatory infertility [5]. OI treatment is associated with ovarian hyper-stimulation and multiple follicular ovulations. As we know, ovulation is a common injurious process associated with an inflammatory response and destruction of ovarian epithelial cells [6, 7]. According to the incessant ovulation and gonadotropin hypothesis, high levels of gonadotropin and excessive ovulation may engage patients into repeated cycles of injury, inducing inflammation and regeneration, which could potentially increase the risk of ovarian cancer by inducing somatic cell mutations [8–10]. Previous studies have debated whether OI could increase the risk of invasive ovarian cancer (IOC) and borderline ovarian tumors (BOT) [11, 12]. Although most studies have concluded that OI does not contribute to the risk of IOC and BOT, some scholars still proposed that OI may be associated with them. Therefore, we performed this updated systematic review and meta-analysis to find out whether exposure to OI treatment significantly increases the risk of IOC and BOT.

## Materials and methods

### Search strategy

The PRISMA guidelines were used for this study. A systematic literature search was then conducted in PubMed, EMBASE, MEDLINE, Google Scholar, Cochrane Library and CNKI, which included records up to January 2022. The main keywords included the following domains of Medical Subject Heading terms: “ ovulation induction “ and “ ovarian cancer “. The retrieval strategy adopted the combination of subject terms and free words. These terms were then combined with “AND” or “OR”. Also, to broaden the search, review articles were used to ensure that all relevant citations were identified and imported.

### Study screening

Two independent researchers (YL and WQQ) simultaneously screened the titles, abstracts and full text of the literature according to the inclusion and exclusion criteria. Any disagreements were discussed and solved by consensus or third-party arbitration (ZS). The inclusion criteria were as follows: (1) Cohort studies and case-control studies with adequate samples; (2) Exposure to ovulation induction drugs such as clomiphene citrate (CC), gonadotrophin (GDT) and gonadotropin-releasing hormone analogs (GnRH-a); (3) Follow-up in the cohort study was sufficiently long to demonstrate treatment differences; (4) The study had a clear description of the exposure to OI drugs and essential information about enrolled patients;(5) The type of cancer included borderline ovarian tumor (BOT) or invasive ovarian cancer (IOC). The exclusion criteria were as follows: (1) Non-English or Non-Chinese literature; (2) Non-human studies; (3) Literature with incomplete data; (4) Duplicate and inaccessible literature.

### Data extraction

Two independent researchers (YL and SJF) performed the data extraction after viewing the complete manuscripts of the eligible literature. Relevant data was input into separate spreadsheets and then cross-checked by each researcher to maintain the quality of the data. The data of bibliography (year and author), study design (sample size, study type, study duration and study location), outcome measures (cancer type and incidence of individual ovarian cancers in group) and other endpoint evaluation (fertility outcome, OI drug type and OI cycles) were extracted from each study. If necessary, discussions with the third-party arbitration (XW) would solve all disputes.

### Quality evaluation

Two researchers (YL and YWN) independently assessed the quality of the literature by using the NOS scale (Newcastle-Ottawa Scale). The main components of the NOS scale included: patient selection, intergroup comparability and outcome measurement [13]. Disagreements were solved by consensus or third-party arbitration (WXL) when they appeared. A total score of more than 6 was considered to be of satisfactory quality [14].

### Statistical analysis

#### Data aggregation and basic meta-analysis

The meta-analysis was performed by using STATA 12.0. Binary variables were evaluated by odds ratio (OR) and its 95% confidence interval (95% CI). *P* < 0.05 was regarded as statistically significant.

Depending on heterogeneity, the appropriate model (random or fixed) was then selected to merge the outcome indicators [15]. The I^2^ value less than 50% were deemed to be low heterogeneity, 51–75% were deemed to be moderate heterogeneity, and greater than 75% were deemed to be high heterogeneity [16]. If the I^2^ value exceeded 50%, the random-effect model was chosen. Otherwise, if the I^2^ value was less than 50%, both the random effects and fixed effects models were acceptable [17].

#### Assessment of publication bias

In principle, funnel plot analyses were performed to accompany meta-analyses involving more 10 studies and to judge the publication bias [18]. If there was no significant publication bias, the funnel plot was supposed to be symmetrical. A complementary approach for funnel plots was to perform Egger’s test to objectively measure bias [19].

#### Details of ethical approval

This meta-analysis was based on the data from published articles and independent of any patient participation. As such, institutional review board (IRB) approval was not required.

## Results

### Study characteristics and quality evaluation

A flowchart detailing the process of identification and inclusion for the target literature was shown in Supplemental Material Fig. 1. Three hundred seven articles were included in the initial screening phase. Of these articles, 42 articles met the criteria for full-text review. Finally, a total of 34 articles were included in the meta-analysis, 14 of which were case-control studies and 20 of which were cohort studies. The final meta-analysis included a total of 3,643,303 participants. All the included literature was of adequate quality. The quality evaluation of the included literature was presented in Supplemental Material Table S1.

### Part I: the risk of ovarian cancer between OI and CT group

Of the 34 studies, 12 reported BOT [12, 20–30] and 30 reported IOC [11, 12, 20, 21, 23–26, 29, 31–51]. Basic information of the included studies was given in Supplemental Material Table S2. For further study, we conducted subgroup analyses to assess the risk of IOC and BOT between groups according to study type.

### The cancer risk between groups in case-control study

**Fig. 1:**
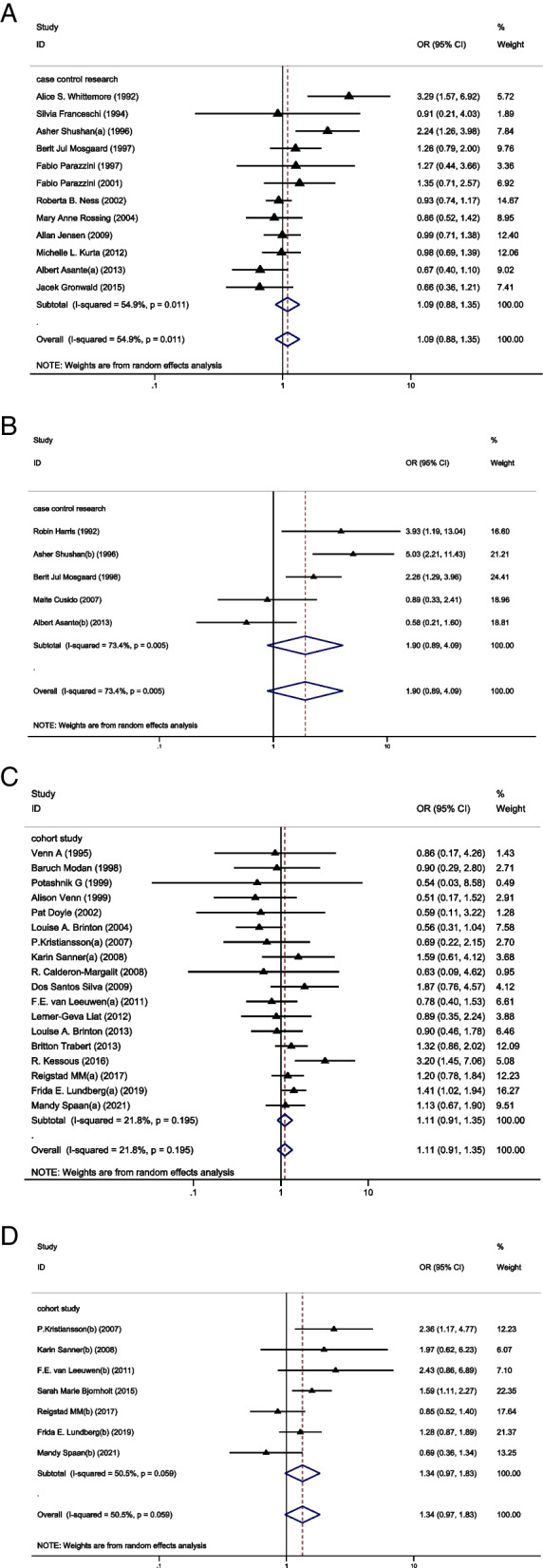
**A** Forest plot of IOC risk between OI group and CT group based on case-control studies; **B** Forest plot of IOC risk between OI group and CT group based on cohort studies; **C** Forest plot of BOT risk between OI group and CT group based on case-control studies; **D** Forest plot of BOT risk between OI group and CT group based on cohort studies

In the subgroup analysis of case-control studies, 12 studies reported IOC [11, 23, 29, 32, 36–43] and 5 studies reported BOT [23, 27–30]. Among these studies, only 1 study showed a significantly higher risk of IOC in the OI group than in the CT group [11] and 3 studies showed a higher risk of BOT in the OI group than in the CT group [28–30]. Pooled result indicated that the risk of IOC (OR = 1.09, 95%CI: 0.88–1.35, I^2^ = 54.9%, Table 1, Fig. 1A) and BOT (OR = 1.90, 95%CI: 0.89–4.09, I^2^ = 73.4%, Table 1, Fig. 1B) did not show significant difference between groups.

**Table 1:** Odd ratios (with confidence intervals) and heterogeneity for each of the cancer risks analysed

Outcome	OR	95% CI	I^2^	Degree of heterogeneity
The risk of IOC between OI and CT group (based on case-control study)	1.09	0.88-1.35	54.9%	Moderate
The risk of BOT between OI and CT group (based on case-control study)	1.90	0.89-4.09	73.4%	Moderate
The risk of IOC between OI and CT group (based on cohort study)	1.11	0.91-1.35	21.8%	Low
The risk of BOT between OI and CT group (based on cohort study)	1.34	0.97-1.83	50.5%	Moderate
The risk of IOC between OI and CT group (in multiparous women)	0.83	0.65-1.05	21.3%	Low
The risk of BOT between OI and CT group (in nulliparous women)	1.17	0.55-2.48	73.5%	Moderate
The risk of IOC between OI and CT group (in nulliparous women)	1.55	0.94-2.57	69.5%	Moderate
The risk of BOT between OI and CT group (in nulliparous women)	**1.49**	**1.03-2.15**	**0%**	**Low**
The risk of IOC between the nulliparous and multiparous women (with ovulation induction treatment)	**3.35**	**2.10-5.34**	**52.2%**	**Moderate**
The risk of BOT between the nulliparous and multiparous women (with ovulation induction treatment)	**2.58**	**1.76-3.79**	**0%**	**Low**
The risk of IOC between OI and CT group (less than 3 ovulation induction cycles)	1.05	0.72-1.52	42.9%	Low
The risk of IOC between OI and CT group (more than 3 ovulation induction cycles)	0.98	0.79-1.22	0%	Low
The risk of IOC between OI and CT group (less than 6 ovulation induction cycles)	0.85	0.64-1.12	0%	Low
The risk of IOC between OI and CT group (more than 6 ovulation induction cycles)	0.88	0.59-1.31	0%	Low
The risk of IOC between OI and CT group (less than 12 ovulation induction cycles)	0.87	0.69-1.10	0%	Low
The risk of IOC between OI and CT group (more than 12 ovulation induction cycles)	0.78	0.49-1.22	0%	Low
The risk of IOC between CC and CT group	1.01	0.88-1.17	0%	Low
The risk of BOT between CC and CT group	1.32	0.79-2.21	72.6%	Moderate
The risk of IOC between GDT and CT group	1.08	0.80-1.44	0%	Low
The risk of BOT between GDT and CT group	1.73	0.88-1.93	54.1%	Moderate
The risk of IOC between HCG and CT group	1.10	0.71-1.71	34.7%,	Low
The risk of BOT between HCG and CT group	1.28	0.71-2.31	56%	Moderate
The risk of IOC between HMG and CT group	1.07	0.44-2.57	71.6%	Moderate
The risk of BOT between HMG and CT group	5.31	0.73-38.72	83.3%	High
The risk of IOC between GnRH-a and CT group	0.49	0.07-3.66	71.9%	Moderate

*CC* Clomiphene citrate, *GDT* Gonadotrophin, *GnRH-a* Gonadotropin-releasing hormone analogues, *HCG* human menopausal gonadotropin, *HMG* human chorionic gonadotropin, *IOC* invasive ovarian cancer, *BOT* borderline ovarian tumor, *OI* ovulation induction group, *CT* control group, *OR* odds ratio, *95%CI* 95% confidence interval

### The cancer risk between groups in cohort study

In the subgroup analysis of cohort studies, 18 studies reported IOC [12, 20, 21, 24–26, 31, 33–35, 44–51] and 7 studies reported BOT [12, 20–22, 24–26]. Of these studies, 3 studies showed a higher risk of IOC [12, 21, 31] in the OI group than in the CT group and 3 studied showed a higher risk of BOT in the OI group than in the CT group [21, 24, 25]. Again, the results showed no significant difference between groups in the incidence of IOC (OR = 1.11, 95%CI: 0.91–1.35, I^2^ = 21.8%, Table 1, Fig. 1C) and BOT (OR = 1.34, 95%CI: 0.97–1.83, I^2^ = 50.5%, Table 1, Fig. 1D).

### Part II: the incidence of ovarian cancer between OI and CT group according to fertility outcome

In this section, we sought to find out whether the multiparous and nulliparous women treated with OI presented an increased risk of ovarian tumors when compared to those who had not been treated with OI. Relevant data were presented in Supplemental Material Table S3.

### The cancer risk between groups in multiparous women

**Fig. 2:**
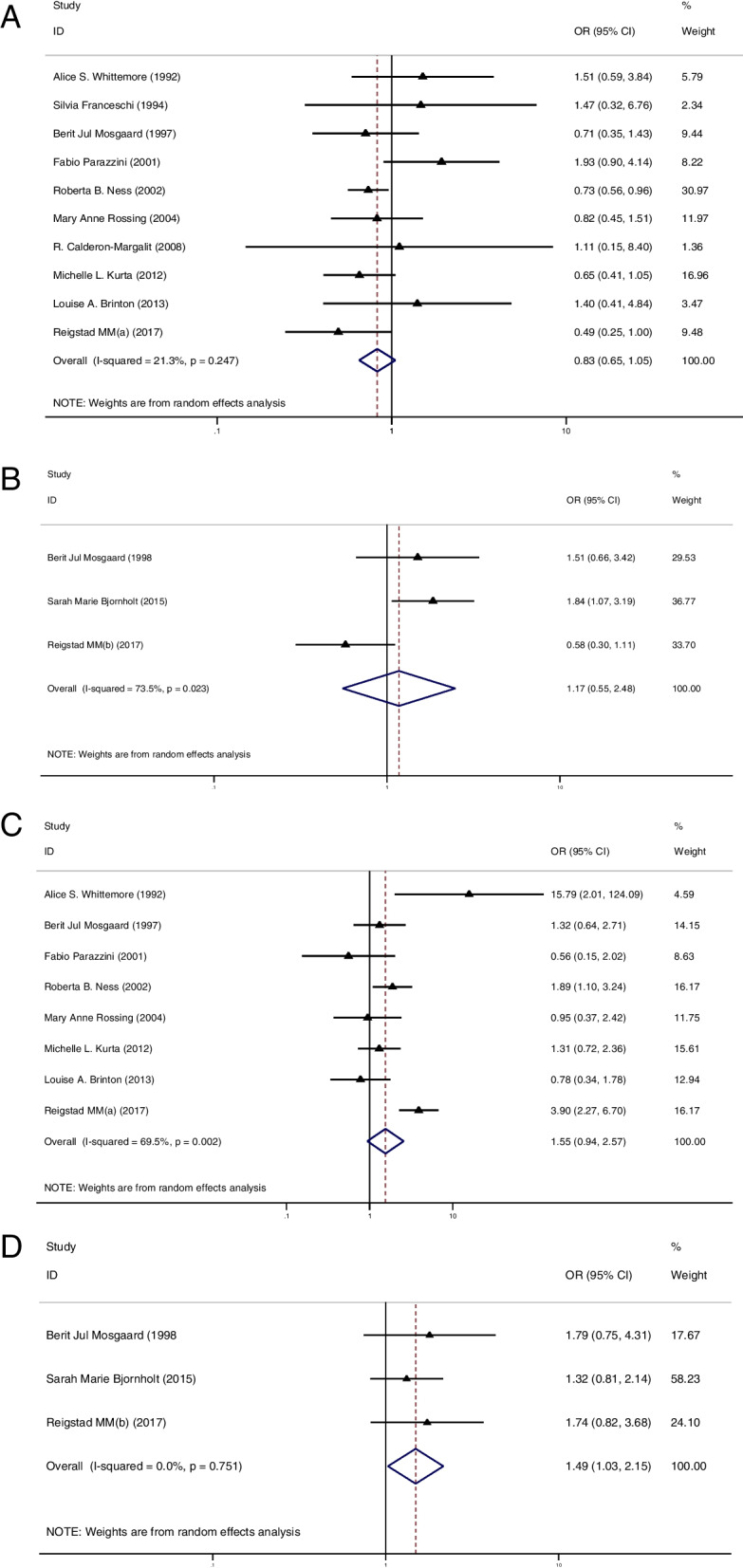
**A** Forest plot of IOC risk between OI group and CT group in multiparous women; **B** Forest plot of BOT risk between OI group and CT group in multiparous women; **C** Forest plot of IOC risk between OI group and CT group in nulliparous women; **D** Forest plot of BOT risk between OI group and CT group in nulliparous women

Firstly, 10 studies of IOC [11, 12, 34, 36–38, 40–42, 50] and 3 studies of BOT [12, 22, 28] analyzed the risk of ovarian cancer in multiparous women with or without OI treatment. None of these studies demonstrated a higher risk for IOC and BOT in the OI group. Pooled result remained consistent, indicating that OI treatment did not increase the risk of IOC (OR = 0.83, 95%CI: 0.65–1.05, I^2^ = 21.3%, Table 1, Fig. 2A) and BOT (OR = 1.17, 95%CI: 0.55–2.48, I^2^ = 73.5%, Table 1, Fig. 2B) in multiparous women.

### The cancer risk between groups in nulliparous women

In the second part, 8 studies of IOC [11, 12, 34, 36, 38, 40–42] and 3 studies of BOT [12, 22, 28] reported the risk of ovarian cancer in nulliparous women with or without OI treatment. Of these studies, only 1 study showed a significantly higher risk of IOC in the OI group than in the CT group [11]. The summarized result for IOC showed no difference in cancer risk between groups (OR = 1.55, 95%CI: 0.94–2.57, I^2^ = 69.5%, Table 1, Fig. 2C). Additionally, none of these studies reported a higher risk of BOT in the OI group. However, after pooled analysis, the risk of BOT appeared to be higher in nulliparous women treated with OI than in those nulliparous women who had not been treated with OI (OR = 1.49, 95%CI: 1.03–2.15, I^2^ = 0%, Table 1, Fig. 2D).

### Part III: the risk of ovarian cancer between the multiparous and nulliparous women in OI group

**Fig. 3:**
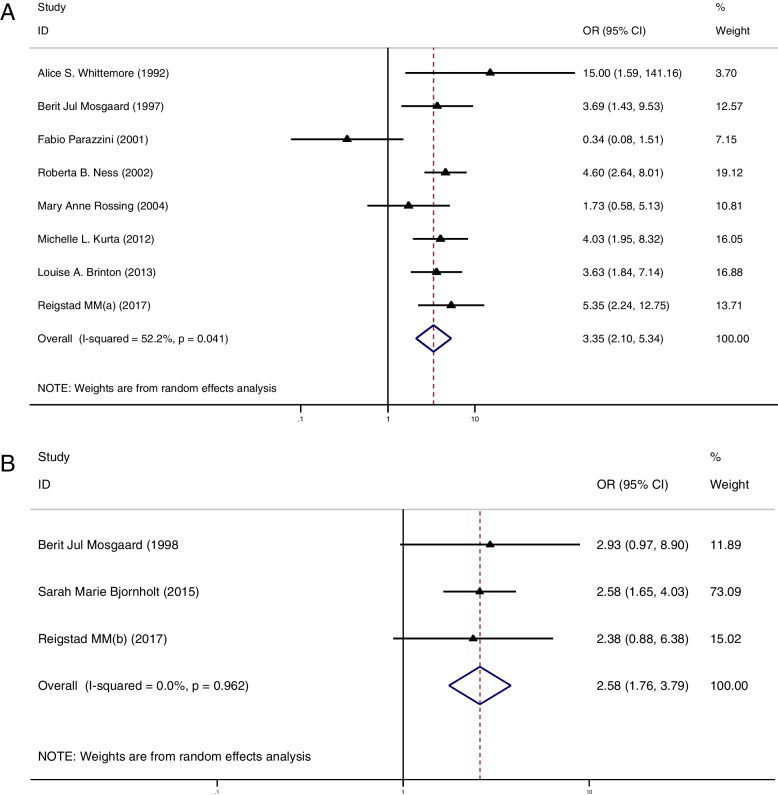
**A** Forest plot of IOC risk between nulliparous and multiparous women with OI treatment; **B** Forest plot of BOT risk between nulliparous and multiparous women with OI treatment

In this chapter, we attempted to figure out the differences in cancer risk between the multiparous and nulliparous woman in the OI group. Relevant data were presented in Supplemental Material Table S4. In total, 8 studies of IOC [11, 12, 34, 36, 38, 40–42] and 3 studies of BOT [12, 22, 28] reported on the risk of ovarian cancer in the nulliparous and multiparous women treated with OI. The summarized results showed a significantly higher risk of IOC (OR = 3.35, 95%CI: 2.10–5.34, I^2^ = 52.2%, Table 1, Fig. 3A) and BOT (OR = 2.58, 95%CI: 1.76–3.79, I^2^ = 0%, Table 1, Fig. 3B) in the nulliparous women treated with OI than in those multiparous women treated with OI.

### Part IV: the relationship between number of OI cycles and cancer risk

Then, we tried to find out whether cancer risk increased with more OI cycles. Totally, 8 studies provided relevant data for IOC [20, 24, 25, 36, 39, 41, 42, 46, 47]. Regrettably, data for BOT were not available for meta-analysis. Relevant data were presented in Supplemental Material Table S5.

**Fig. 4:**
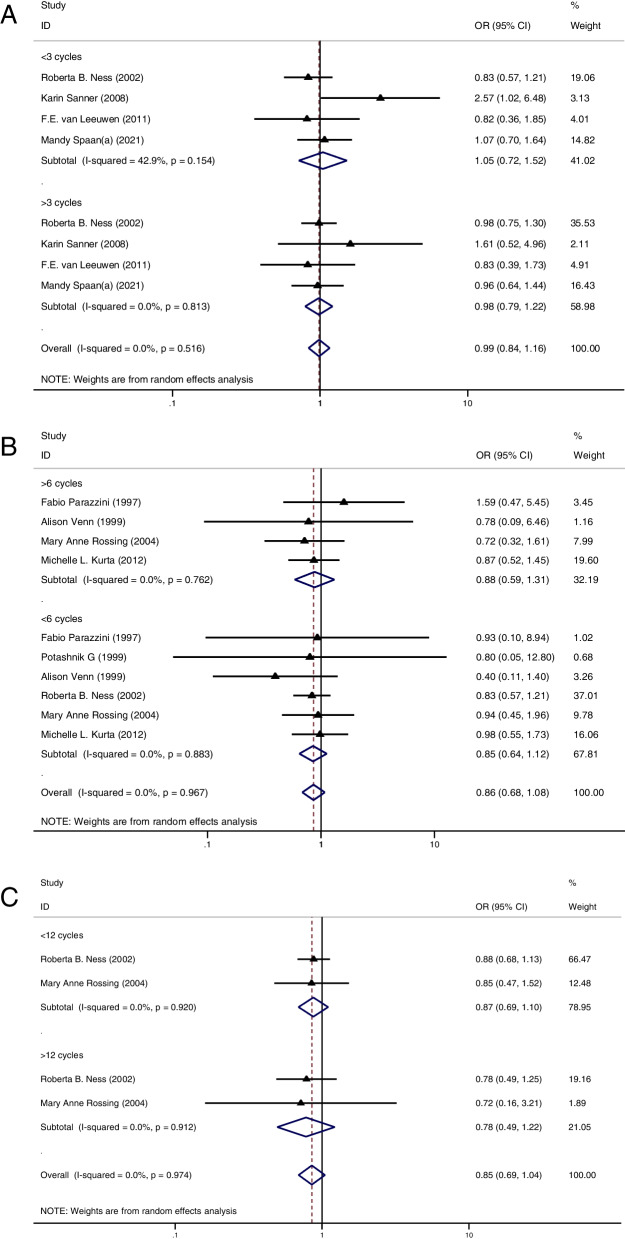
**A** Forest plot of IOC risk between OI group and CT group based on a cut-off value of 3 cycles; **B** Forest plot of IOC risk between OI group and CT group based on a cut-off value of 6 cycles; **A** Forest plot of IOC risk between OI group and CT group based on a cut-off value of 12 cycles

Using a cut-off of 3 cycles, we did not find a higher cancer risk in those women who received less than 3 cycles when compared to the CT group (OR = 1.05, 95%CI: 0.72–1.52, I^2^ = 42.9%, Table 1, Fig. 4A). Meanwhile, we found a similar result in those women who received more than 3 cycles (OR = 0.98, 95%CI: 0.79–1.22, I^2^ = 0%, Table 1, Fig. 4A). Using 6 cycles as a cut-off, those women who received less than 6 cycles did not present an increased cancer risk when compared to the CT group (OR = 0.85, 95%CI: 0.64–1.12, I^2^ = 0%, Table 1, Fig. 4B) and a similar result was found in those women who received more than 6 OI cycles (OR = 0.88, 95%CI: 0.59–1.31, I^2^ = 0%, Table 1, Fig. 4B). Lastly, using 12 cycles as a cut-off, we did not find a significantly increased cancer risk in those women who received less than 12 cycles when compared to the CT group (OR = 0.87, 95%CI: 0.69–1.10, I^2^ = 0%, Table 1, Fig. 4C). Also, a similar result was found in those women who received more than 12 OI cycles (OR = 0.78, 95%CI: 0.49–1.22, I^2^ = 0%, Table 1, Fig. 4C).

### Part V: the relationship between specific OI treatment and cancer risk

At last, we wished to find out whether specific OI drugs were associated with an increased cancer risk. For further study, we divided the subjects into three groups according to the type of OI drug. These were the clomiphene citrate group (CC), the gonadotrophin group (GDT) and the gonadotropin-releasing hormone analog group (GnRH-a). Relevant data were provided in Supplemental Material Table S6.

### The relationship between CC and cancer risk

**Fig. 5:**
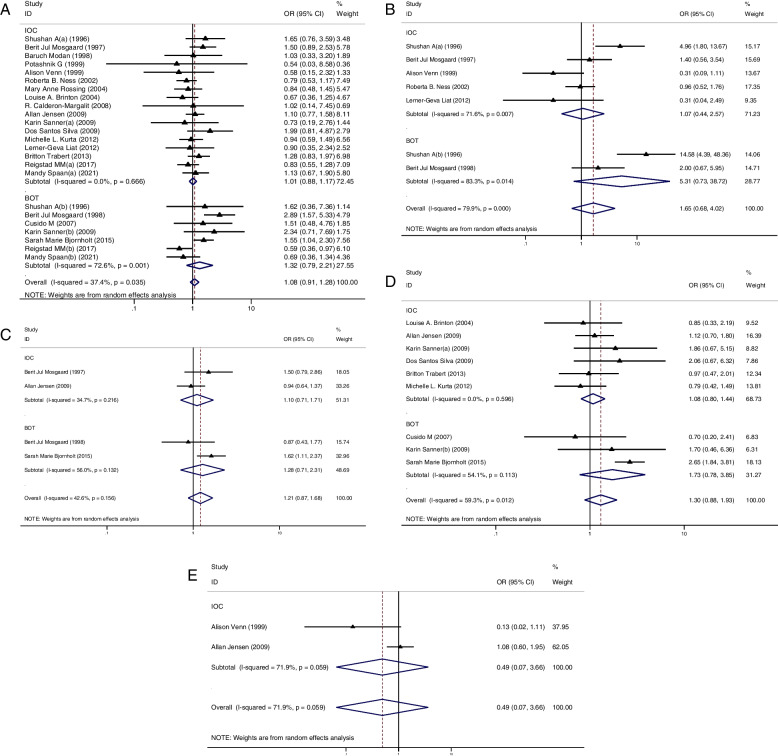
**A** Forest plot of IOC and BOT risk between CC group and CT group; **B** Forest plot of IOC and BOT risk between HMG group and CT group; **C** Forest plot of IOC and BOT risk between HCG group and CT group; **D** Forest plot of IOC and BOT risk between GDT group and CT group; **E** Forest plot of IOC risk between GnRH group and CT group

We firstly analyzed the relationship between CC and cancer risk. This part of analysis included 17 studies of IOC [25, 29, 35, 36, 38, 41–43, 45–47, 50, 51] and 7 studies of BOT [12, 20, 22, 25, 27–29]. Only 1 study reported a higher cancer risk in the CC group than in the CT group [12]. However, pooled results showed that the risk of IOC (OR **=** 1.01, 95%CI: 0.88–1.17, I^2^ = 0%, Table 1, Fig. 5A) and BOT (OR = 1.32, 95%CI: 0.79–2.21, I^2^ = 72.6%, Table 1, Fig. 5A) were not significantly higher in the CC group when compared to the CT group.

### The relationship between GDT and cancer risk

Secondarily, we focused our attention on GDT and performed a subgroup analysis in this section. GDTs mainly consisted of human menopausal gonadotropin (HMG) and human chorionic gonadotropin (HCG). However, some studies did not further categorized GDT. For IOC, there were 5 studies of HMG [29, 35, 38, 41, 47], 2 studies of HCG [38, 43] and 6 studies of unclassified GDT [25, 33, 36, 43, 49, 51]. Only 1 study reported a higher cancer risk in HMG group than in the CT group [29]. Nevertheless, pooled results indicated that HMG (OR = 1.07, 95%CI: 0.44–2.57, I^2^ = 71.6%, Table 1, Fig. 5B), HCG (OR = 1.10, 95%CI: 0.71–1.71, I^2^ = 34.7%, Table 1, Fig. 5C) and unclassified GDT (OR = 1.08, 95%CI: 0.80–1.44, I^2^ = 0%, Table 1, Fig. 5D) did not increase the cancer risk.

While for BOT, 2 studies of HMG [28, 29], 2 studies of HCG [22] and 3 studies of GDT [22, 25, 27] were included in this part of analysis. Consistently, we found similar results that HMG (OR = 5.31, 95%CI: 0.73–38.72, I^2^ = 83.3%, Table 1, Fig. 5B), HCG (OR = 1.28, 95%CI: 0.71–2.31, I^2^ = 56%, Table 1, Fig. 5C) and unclassified GDT (OR = 1.73, 95%CI: 0.88–1.93, I^2^ = 54.1%, Table 1, Fig. 5D) did not increase tumor risk.

### The relationship between GnRH-a and cancer risk

Thirdly, we only found 2 studies which provided analyzable data on the relationship between the risk of IOC and GnRH-a [43, 47]. Along the same lines, we did not find an increased risk of IOC (OR = 0.49, 95%CI: 0.07–3.66, I^2^ = 71.9%, Table 1, Fig. 5E) in the GnRH-a group when compared to the CT group. However, it was regrettable that another meta-analysis focusing on the relationship between the risk of BOT and GnRH-a could not be performed due to lack of data.

### Publication bias

In our analysis, funnel plot analysis and Egger regression analysis were performed to judge the publication bias of the included studies. Neither the funnel plot (Supplemental Material Fig. 2A) nor the Egger test showed evidence of publication bias in our analysis (Supplemental Material Fig. 2B).

## Discussion

The following points are currently discussed regarding the possible induction of ovarian cancer with the use of OI drugs: (1) the incessant ovulation hypothesis stated that ovarian epithelium could be destroyed and repaired during uninterrupted ovulation. When a sufficient amount of damage is caused, malignant transformation of ovarian epithelial cells will be triggered [8]. Furthermore, cancer risk had been found to decrease with increasing numbers of pregnancies and live births, longer duration of breastfeeding and use of oral contraceptives [52–57]. The effects of these anovulation factors confirmed the above observations. Thus, it is thought that the number of ovulatory cycles during the lifetime was associated with ovarian cancer risk, this finding has been observed in several animal models and epidemiological studies [58–62]. (2) The gonadotropin hypothesis suggested that excess gonadotropins could hyper-stimulate the ovaries and induce estrogen production. The amount of estrogen secreted in one gonadotropin-stimulated cycle was equivalent to the total production of natural cycles over a two-year period [63]. Meanwhile, there appeared to be growing evidence that estrogen conferred increased ovarian cancer risk [64–66]. Thus, gonadotropin-induced elevated estrogen levels might promote the malignant transformation of normal ovarian epithelium [63, 67]. The above hypotheses had been tested in hen models but not in humans [68, 69]. Therefore, speculation regarding the relationship between the use of ovulation induction drug use and ovarian cancer development continues.

Research in this area was based on cohort studies, case series and case-control studies. And, there were still no randomized controlled trials regarding the relationship between ovulation inducing drugs and ovarian cancer due to ethical issues, the relatively low incidence of ovarian cancer and recall bias after ovulation induction [70]. This updated systematic review and meta-analysis was based on cohort studies and case-control studies. Some of included studies provided supportive evidence that OI treatment might increase the risk of IOC. However, according to subgroup analysis based on study type, we found no convincing evidence that OI treatment could induce an increased risk of IOC. Compared with previous systematic reviews [71, 72], we expanded our search to include more recent studies in our analysis and obtained consistent results.

BOTs are morphologically similar to IOCs and follow a similar pathogenesis [73, 74]. As the etiology of BOT was still unknown, it was difficult to explain the possible causal relationship between infertility and OI drugs. In our analysis, no significant increased risk of BOT was found following OI treatment, which appeared to contradict the increasing risk of BOT reported by Barcroft et.al (OR = 1.69, 95%CI: 1.27–2.25). With more studies included in our pooled analysis, we used further subgroup analyses based on study type to circumvent the heterogeneity issues caused by retrospective studies. Ultimately, the results of subgroup analysis were highly consistent in that OI treatment did not increase the risk of BOT.

In addition, during the review of the literature, we found several studies supporting that OI treatment could induce an increased risk of ovarian cancer in the nulliparous women [11, 12]. A cumulative analysis conducted by Whittemore et.al indicated an increased risk of ovarian cancer (OR = 27.0, 95%CI: 2.3–315.6) in the nulliparous women who ever received OI treatment. Reigstad et.al also noted a greater increased risk of ovarian cancer (HR 2.49, 95% CI 1.30 to 4.78) in the nulliparous women treated with OI. As we know, parity was known as an established protective factor for ovarian cancer [75]. Previous studies have shown that the greatest reduction in ovarian cancer risk was associated with the first pregnancy and each subsequent pregnancy could also reduce the risk of ovarian cancer [11, 75, 76]. This protective mechanism has been attributed to anovulation, reduced gonadotropin production and increased progesterone levels [77]. Hence, whether OI treatment would increase the ovarian cancer risk in nulliparous and multiparous women remained controversial. Therefore, we conducted supplementary analyses based on fertility outcomes in light of the above questions. Among the multiparous women, we did not find a higher risk of IOC and BOT in the OI group than in the CT group. Similarly, among the nulliparous women, OI treatment also did not increase the risk of IOC. However, an increased risk of BOT was found in the nulliparous women treated with OI when compared to those nulliparous women who had not been treated with OI. Nonetheless, none of these included studies initially reported a higher risk of BOT in nulliparous women treated with OI. Based on a review of the included studies, this finding might be due to a lack of ovulatory pause caused by pregnancy and exposure to ovarian hyper-stimulation [8–10, 12]. Notably, the BOTs were generally seen in younger women [78–81]. Hence, the above association might also be due to a diagnostic bias occurring in young nulliparous women who might pursue medical attention and undergo intensive monitoring [41].

Rodriguez et.al previously found that infertility itself might increase ovarian cancer risk without concomitant exposure to OI drugs [82]. A current meta-analysis based on nine prospective cohort studies also suggested that infertility in women was associated with an increased risk of ovarian cancer [83]. Moreover, a number of diseases that cause infertility, including polycystic ovary syndrome (PCOS) and endometriosis, had been found to be associated with ovarian cancer development. Previous studies had indicated that the genetic and epigenetic profile of patients with PCOS was similar to that of ovarian cancer [84]. Further, the risk of ovarian cancer, particularly serous borderline ovarian tumor, was shown to be increased in patients with PCOS [85–87]. We also found that ovarian clear cell carcinoma and endometrioid carcinoma were most often associated with ovarian endometriosis in previous studies [88, 89]. Thus, infertility itself might be an independent risk factor for ovarian cancer [90]. In parallel, whether there existed a difference in cancer risk between nulliparous and multiparous women treated with OI was under discussion, as it was difficult to separate OI treatment from infertility as a risk factor for ovarian cancer. In our analysis, we used OI exposure as a control variable to evaluate the relationship between infertility and ovarian cancer and found that the nulliparous women treated with OI showed a higher risk of IOC and BOT than those multiparous women treated with OI. Nieto et.al performed a retrospective study of ovarian cancer in first-degree relatives of infertile patients and showed an increased risk of ovarian cancer in infertile patients who failed to conceive despite receiving OI treatment, which supported our findings [91]. Rizzuto et.al also noted that the risk of BOT was slightly higher in nulliparous women treated with OI than in multiparous women [72]. In summary, we believed that there was a necessity to conduct a rigorous medical follow-up in those nulliparous patients treated with OI. Consistent with previous studies, the vast majority of patients were found within 5 years after ovulation induction [92–98]. In our analysis, the included cohort studies had a follow-up period of more than 5 years, which in our opinion is sufficient to detect ovarian cancer. Therefore, follow-up periods longer than 5 years should be considered.

Indeed, it would be arbitrary to diagnose the relationship between OI and ovarian cancer solely based on the history of OI exposure. According to incessant ovulation hypothesis, more ovulatory cycles appeared to be associated with a higher risk of developing ovarian cancer [75, 99, 100]. Whether such a cumulative effect exists remained controversial. After reviewing previous studies, we found no meta-analysis reported an association between OI cycles and the risk of ovarian cancer. Thereby, we performed a further subgroup analysis based on OI cycles, which was the focal point of our analysis. In our analysis, we used 3, 6 and 12 OI cycles as cut-off points, respectively. Compared to the control population, we found no correlation between increasing OI cycles and increased cancer risk. Unfortunately, the data for BOT in this aspect were not available for meta-analysis.

In accession, several studies had reported the risk of individual ovarian cancers due to specific OI drug exposure [12, 29, 101]. Consequently, for further study, we performed subgroup analyses according to the type of OI drug to assess whether specific OI drugs would increase the risk of ovarian cancer. CC was the most common drug to induce ovulation, especially in patients with ovulatory disturbances [102]. Reigstad et.al reported an increased risk of cancer in nulliparous women exposed to CC (HR = 2.5, 95%CI: 1.3–4.8). Rossing et.al also reported an increased ovarian tumor risk in women exposed to CC (SIR = 2.5, 95%CI: 1.3–4.5). A current meta-analysis conducted by Barcroft et.al supported the view mentioned above, which concluded that the exposure to CC was associated with a significant increased cancer risk (OR = 1.40, 95%CI: 1.10–1.77). However, in our meta- analysis, we included additional studies but did not find an increased cancer risk in those women exposed to CC. GDTs were also commonly used in women with proven hypopituitarism and in women who were not sensitive to CC [103, 104]. Shan et.al reported a slight increased ovarian cancer risk in women exposed to HMG (OR = 3.95, 95%CI: 1.3–12.2). While in our study, we found that GDTs were not associated with an increased risk of IOC and BOT. GnRH-a was introduced in anovulatory women, which could reproduce spontaneous menstrual cycle and induce ovulation [105, 106]. Our findings indicated that GnRH-a did not increase the risk of IOC. Due to the lack of the data on BOT risk in women exposed to GnRH-a, further meta-analysis could not be performed. In summary, CC, GDT and GnRH-a were proven to be safe for OI treatment without increasing ovarian tumor risk.

Most of our findings were generally consistent with previous studies on this topic [71, 72, 107, 108]. A new study was included in this latest update of the systematic review and meta-analysis compared to previous studies in this area. This study provides new data on the risk of BOT and IOC to CC exposure. To assess the impact of the latest studies on the outcome of this update, an additional sensitivity analysis was conducted. The sensitivity analysis without the latest study did not change the results that exposure to CC did not increase the risk of IOC (OR = 1.05, 95%CI: 0.89–1.21) and BOT (OR = 1.73, 95%CI: 0.96–2.50). And this latest study made the results more reliable. The results of the sensitivity analysis were shown in Supplemental Material Table S7. To summarize, OI treatment was relatively safe and cancer risk was not increased more cycles of OI and specific OI drugs. However, for those nulliparous women treated with OI, they appeared to have a higher tumor risk. Therefore, rigorous monitoring and sufficiently long follow-up were necessary for these women.

### Strengths and limitations of the study

This study included 34 studies from around the world and provided an up-to-date meta-analysis to explore the potential impact of OI treatment on ovarian cancer risk. The inclusion and exclusion criteria for this systematic review and meta-analysis had been made more rigorous. In addition, the included studies were updated and the process of meta-analysis was made more rigorous. In our analysis, lessons learned from previous studies were incorporated and further subgroup analyses were conducted based on study type, tumor type, parity, OI cycle and specific OI drugs. Of note, this meta-analysis was the first study to evaluate the relationship between the OI cycles and ovarian cancer.

However, our study still had some objective shortcomings. Firstly, further work should focus more attention on patient demographics and specific data including drug combinations, cycles of use, use dosage and administration methods. Secondly, loss of follow-up existed in included studies in our analysis and retrospective studies were always considered to be lower quality evidence due to the presence of recall bias. We needed more large and long-term prospective cohort studies with careful follow-up. Thus, follow-up process needed to be improved. Last but not least, the formation of symbiotic relationships between cancer registries and fertility services should be encouraged to link fertility data with cancer information. Communication and collaboration between fertility services should also be encouraged in order to collect adequate data. We believe that further exploration in this area will facilitate the further development of reproductive science.

## Conclusions

OI treatment did not increase risk of ovarian cancer, regardless of treatment regimen and treatment cycle. However, nulliparous women treated with OI might have an increased risk of BOT compared to the nulliparous women not treated with OI. Meanwhile, nulliparous women treated with OI appeared to have a higher risk of IOC and BOT than multiparous women treated with OI. In view of the above, OI treatment was relatively safe but those nulliparous women treated with OI must be followed up rigorously.

## Supplementary Information

**Additional file 1: Supplemental Material Fig. 1.** The flowchart of systematic search and screening process.

**Additional file 2: Supplemental Material Fig. 2.** (A) Funnel plot of all the included studies; (B) Egger’s regression test of all the included studies.

**Additional file 3: Supplementary Table S1a.** Quality evaluation of case-control study assessed by the Newcastle-Ottawa Scale. **Supplementary Table S1b.** Quality evaluation of cohort study assessed by the Newcastle-Ottawa Scale.

**Additional file 4: Supplementary Table S2**. ovarian cancer study characteristics.

**Additional file 5: Supplementary Table S3a**. Ovarian tumor in the nulliparous women. **Supplementary Table S3b**. Ovarian tumor in the multiparous women.

**Additional file 6: Supplementary Table S4**. Ovarian tumor between the nulliparous and multiparous group.

**Additional file 7: Supplementary Table S5a**. Ovarian tumor and stimulation cycles(< 3 cycles). **Supplementary Table S5b**. Ovarian tumor and stimulation cycles(≥3 cycles). **Supplementary Table S5c**. Ovarian tumor and stimulation cycles(< 6 cycles). **Supplementary Table S5d**. Ovarian tumor and stimulation cycles(≥6 cycles). **Supplementary Table S5e**. Ovarian tumor and stimulation cycles(< 12 cycles). **Supplementary Table S5f**. Ovarian tumor and stimulation cycles(≥12 cycles).

**Additional file 8: Supplementary Table S6a**. Ovarian tumor in women who ever received CC. **Supplementary Table S6b**. Ovarian tumor in women who ever received HMG. **Supplementary Table S6c**. Ovarian tumor in women who ever received HCG. **Supplementary Table S6d**. Ovarian tumor in women who ever received GDT. **Supplementary Table S6e**. Ovarian tumor in women who ever received GnRH-a.

**Additional file 9: Supplementary Table S7**. Sensitivity analyses for the novel study during this update.

## Data Availability

This meta-analysis was based on the data from the published articles and independent of any patient involvement. All the data will be made available to the editors of the journal for review or query upon.

## Abbreviations

OI: Ovulation induction
OR: Odds ratio
BOT: Borderline ovarian tumor
IOC: Invasive ovarian cancer
CC: Clomiphene citrate
GDT: Gonadotrophin
GnRH-a: Gonadotropin-releasing hormone analogues
