# Supplementary material for: Ovulation induction drug and ovarian cancer: an updated systematic review and meta-analysis
Source: J Ovarian Res. 2023 Jan 24;16:22. doi: 10.1186/s13048-022-01084-z (PMC9872323; doi:10.1186/s13048-022-01084-z)
Supplement: Supplementary file 1 — Additional file 1: Supplemental Material Fig. 1. The flowchart of systematic search and screening process. [file 13048_2022_1084_MOESM1_ESM.pdf]

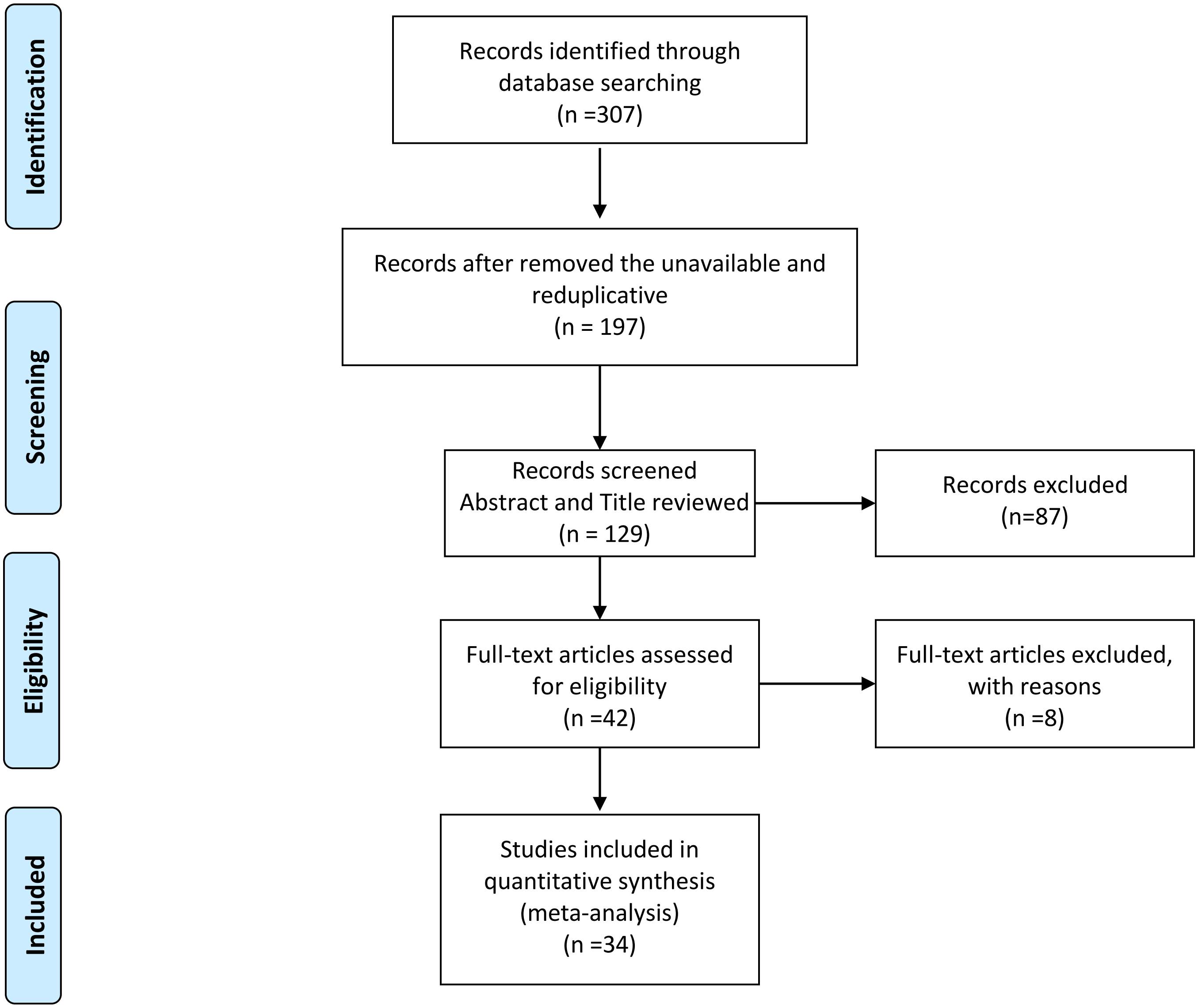
Identification
Screening
Eligibility
Included
Records identified through database searching (n =307)
Records after removed the unavailable and reduplicative (n = 197)
Records screened Abstract and Title reviewed (n = 129)
Records excluded (n=87)
Full-text articles assessed for eligibility (n =42)
Full-text articles excluded, with reasons (n =8)
Studies included in quantitative synthesis (meta-analysis) (n =34)
