# Supplementary figures and images for: Ovulation induction drug and ovarian cancer: an updated systematic review and meta-analysis
Source: J Ovarian Res. 2023 Jan 24;16:22. doi: 10.1186/s13048-022-01084-z (PMC9872323; doi:10.1186/s13048-022-01084-z)

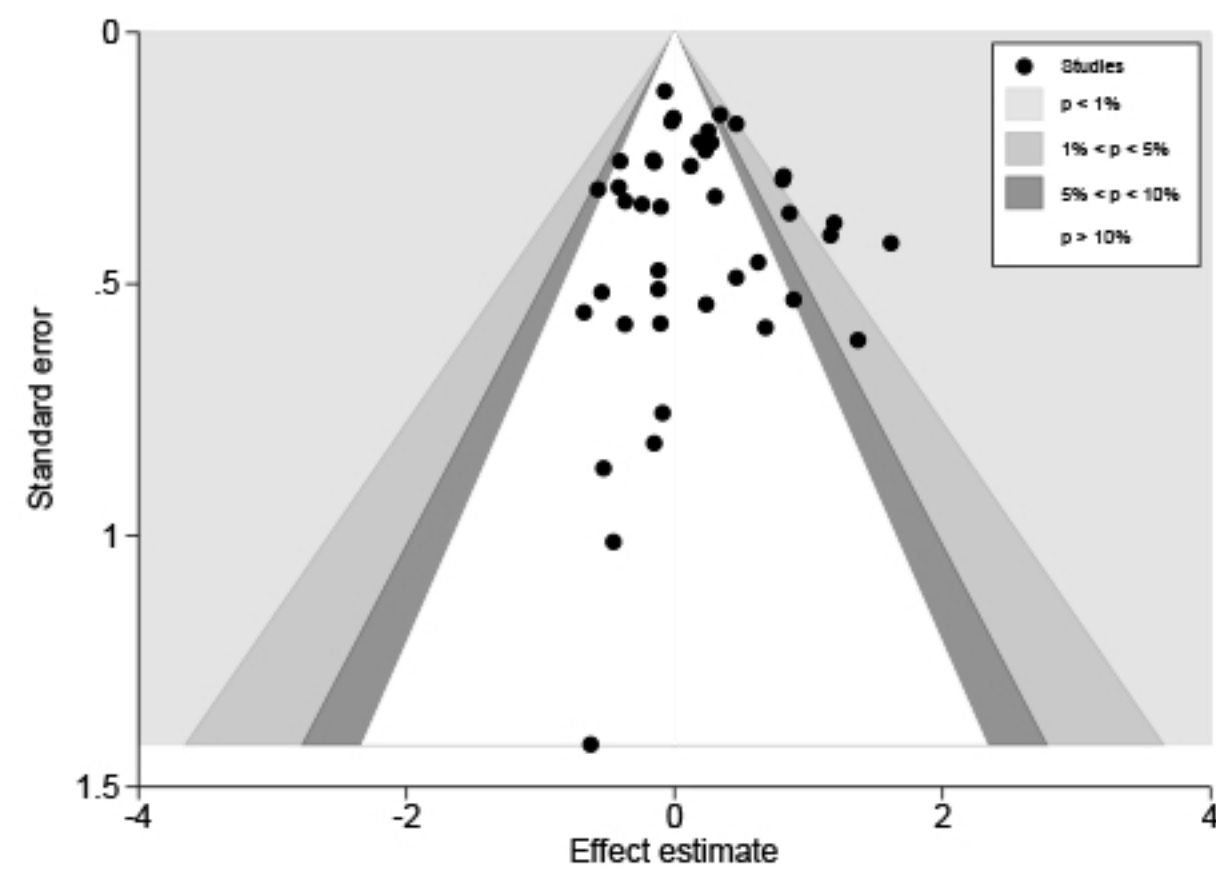

A

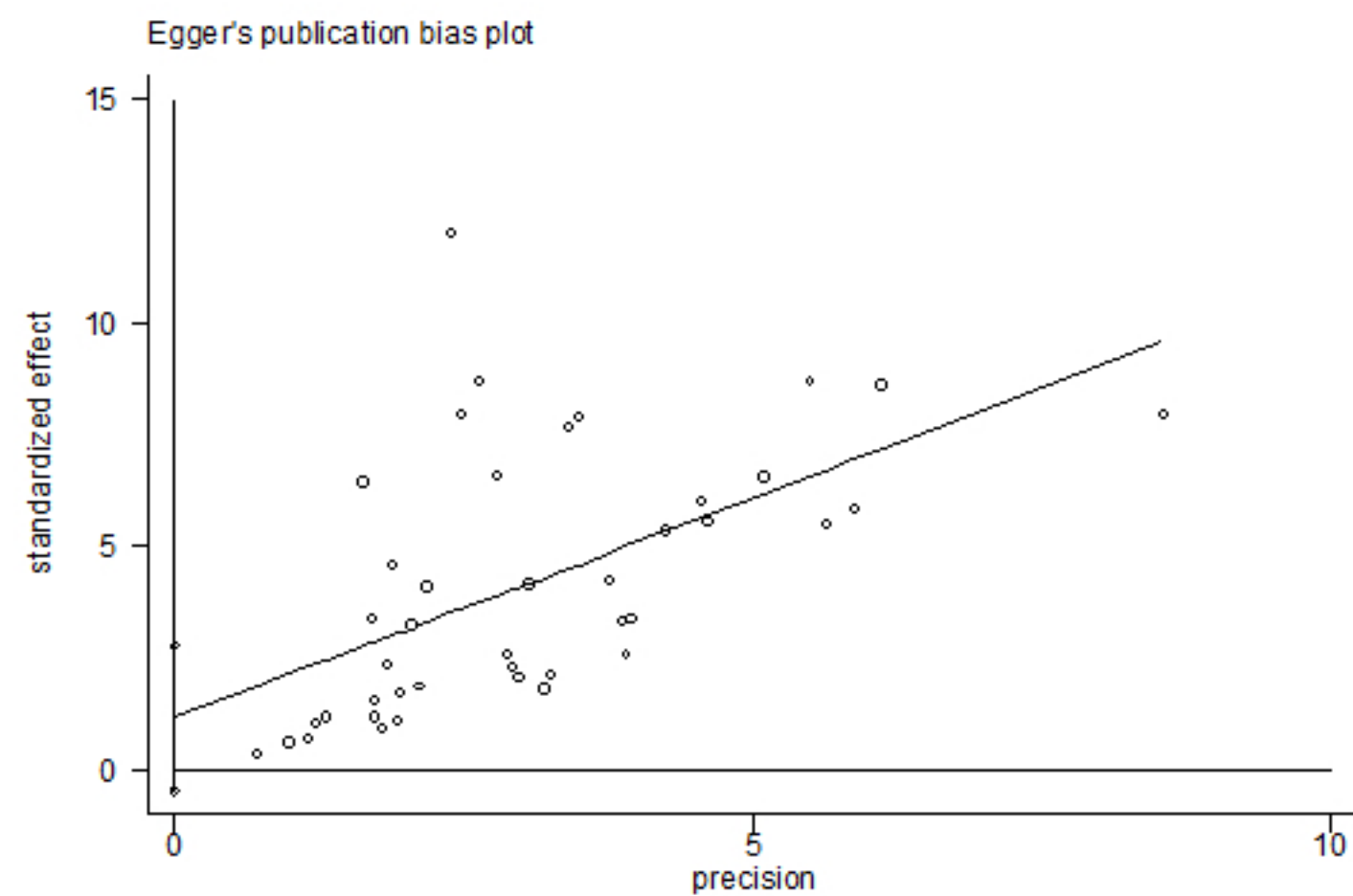

B

Supplement: Supplementary file 2 — Additional file 2: Supplemental Material Fig. 2. (A) Funnel plot of all the included studies; (B) Egger’s regression test of all the included studies. [file 13048_2022_1084_MOESM2_ESM.pdf]
