# Supplementary material for: Ovulation induction drug and ovarian cancer: an updated systematic review and meta-analysis
Source: J Ovarian Res. 2023 Jan 24;16:22. doi: 10.1186/s13048-022-01084-z (PMC9872323; doi:10.1186/s13048-022-01084-z)
Supplement: Supplementary file 3 — Additional file 3: Supplementary Table S1a. Quality evaluation of case-control study assessed by the Newcastle-Ottawa Scale. Supplementary Table S1b. Quality evaluation of cohort study assessed by the Newcastle-Ottawa Scale. [file 13048_2022_1084_MOESM3_ESM.docx]

Supplementary Table S1a: Quality evaluation of case-control study assessed by the Newcastle-Ottawa Scale.

| Author | Case definition | Representativeness | Control selection | Control definition | Comparability | Ascertainment of exposure | Same method of ascertainment for cases and controls | Non-Response rate | Total score |
| --- | --- | --- | --- | --- | --- | --- | --- | --- | --- |
| Robin Harris  (1992) | 1 | 1 | 1 | 1 | 1 | 1 | 1 | 0 | 7 |
| Alice S. Whittemore  (1992) | 1 | 1 | 1 | 1 | 1 | 1 | 1 | 0 | 7 |
| Silvia Franceschi  (1994) | 1 | 1 | 0 | 1 | 2 | 1 | 1 | 0 | 7 |
| Shushan A  (1996) | 1 | 1 | 1 | 1 | 2 | 1 | 1 | 1 | 9 |
| Berit Jul Mosgaard  (1997) | 1 | 1 | 1 | 1 | 2 | 1 | 1 | 1 | 9 |
| Fabio Parazzini  (1997) | 1 | 1 | 0 | 1 | 1 | 1 | 1 | 1 | 7 |
| Berit Jul Mosgaard  (1998) | 1 | 1 | 1 | 1 | 2 | 1 | 1 | 1 | 9 |
| Fabio Parazzini  (2001) | 1 | 1 | 0 | 1 | 1 | 1 | 1 | 0 | 6 |
| Roberta B. Ness  (2002) | 1 | 1 | 1 | 1 | 2 | 1 | 1 | 0 | 8 |
| Mary Anne Rossing  (2004) | 1 | 1 | 1 | 1 | 2 | 1 | 1 | 1 | 9 |
| Maite Cusido  (2007) | 1 | 1 | 0 | 1 | 1 | 1 | 1 | 0 | 6 |
| Michelle L. Kurta  (2013) | 1 | 1 | 1 | 1 | 1 | 1 | 1 | 0 | 7 |
| Albert Asante  (2013) | 1 | 1 | 1 | 1 | 2 | 1 | 1 | 0 | 8 |
| Jacek Gronwald  (2016) | 1 | 1 | 1 | 1 | 1 | 1 | 1 | 1 | 8 |

Supplementary Table S1b: Quality evaluation of cohort study assessed by the Newcastle-Ottawa Scale.

| Author | Representativeness of the exposed cohort | Selection of the non- exposed cohort | Ascertainment of exposure | Demonstration that outcome of interest was not present at start of study | Comparability | Assessment of outcome | follow-up long enough for outcomes to occur | Adequacy of follow up of cohorts | Total score |
| --- | --- | --- | --- | --- | --- | --- | --- | --- | --- |
| Venn A  (1995) | 1 | 1 | 1 | 1 | 1 | 1 | 1 | 0 | 7 |
| Baruch Modan  (1998) | 1 | 1 | 1 | 1 | 1 | 1 | 1 | 1 | 8 |
| Potashnik G  (1999) | 1 | 1 | 1 | 1 | 1 | 1 | 1 | 1 | 8 |
| Alison Venn  (1999) | 1 | 1 | 1 | 1 | 1 | 1 | 1 | 1 | 8 |
| Pat Doyle  (2002) | 1 | 1 | 1 | 1 | 1 | 1 | 1 | 1 | 8 |
| Louise A. Brinton  (2004) | 1 | 1 | 1 | 1 | 1 | 1 | 1 | 0 | 7 |
| P.Kristiansson  (2007) | 1 | 1 | 1 | 1 | 1 | 1 | 1 | 1 | 8 |
| Allan Jensen  (2009) | 1 | 1 | 1 | 1 | 1 | 1 | 1 | 1 | 8 |
| R.Calderon-Margalit  (2009) | 1 | 1 | 1 | 1 | 1 | 1 | 1 | 1 | 8 |
| Santos Silva  (2009) | 1 | 1 | 1 | 1 | 1 | 1 | 1 | 1 | 8 |
| Karin Sanner  (2009) | 1 | 1 | 1 | 1 | 1 | 1 | 1 | 1 | 8 |
| F.E. van Leeuwen  (2011) | 1 | 1 | 1 | 1 | 1 | 1 | 1 | 0 | 7 |
| Lerner-Geva Liat  (2012) | 1 | 1 | 1 | 1 | 1 | 1 | 1 | 1 | 8 |
| Louise A. Brinton  (2013) | 1 | 1 | 1 | 1 | 2 | 1 | 1 | 1 | 9 |
| Britton Trabert  (2013) | 1 | 1 | 1 | 1 | 1 | 1 | 1 | 0 | 7 |
| Sarah Marie Bjornholt  (2015) | 1 | 1 | 1 | 1 | 1 | 1 | 1 | 1 | 8 |
| R. Kessous  (2016) | 1 | 1 | 1 | 1 | 1 | 1 | 1 | 0 | 7 |
| Reigstad MM  (2017) | 1 | 1 | 1 | 1 | 2 | 1 | 1 | 0 | 8 |
| Frida E. Lundberg  (2019) | 1 | 1 | 1 | 1 | 2 | 1 | 1 | 1 | 9 |
| Mandy Spaan  (2021) | 1 | 1 | 1 | 1 | 2 | 1 | 1 | 1 | 9 |
