# Supplementary material for: Ovulation induction drug and ovarian cancer: an updated systematic review and meta-analysis
Source: J Ovarian Res. 2023 Jan 24;16:22. doi: 10.1186/s13048-022-01084-z (PMC9872323; doi:10.1186/s13048-022-01084-z)
Supplement: Supplementary file 4 — Additional file 4: Supplementary Table S2. ovarian cancer study characteristics. [file 13048_2022_1084_MOESM4_ESM.docx]

Supplementary Table S2: ovarian cancer study characteristics.

| Author | Year | Study location | Study type | Tumor type | Study time  (Duration) | Numbers of volunteer within subgroups | Endpoint evaluation |
| --- | --- | --- | --- | --- | --- | --- | --- |
| Robin Harris(*1*) | 1992 | America | case control research | BOT | 1974-1986 | N_OI-OT_=4；N_OI-NOR_=9  N_CT-OT_=84；N_CT-NOR_=743 | OR=4.00,95%CI（1.1-14.9） |
| Alice S. Whittemore(*2*) | 1992 | America | case control research | IOC | 1956-1986 | N_OI-OT_=20；N_OI-NOR_=11  N_CT-OT_=602；N_CT-NOR_=1090 | OR=2.8,95%CI（1.3-6.1） |
| Silvia Franceschi(*3*) | 1994 | Italy | case control research | IOC | 1992-1993 | N_OI-OT_=2；N_OI-NOR_=15  N_CT-OT_=193；N_CT-NOR_=1324 | OR=0.73,95%CI（0.16-3.30） |
| Venn A(*4*) | 1995 | Australia | cohort study | IOC | 1978-1992 | N_OI-OT_=3；N_OI-NOR_=5522  N_CT-OT_=3；N_CT-NOR_=4746 | RR=1.45,95%CI（0.28-7.55） |
| Shushan A(a)(*5*) | 1996 | Israel | case control research | IOC | 1990-1993 | N_OI-OT_=24；N_OI-NOR_=29  N_CT-OT_=140；N_CT-NOR_=379 | OR=1.31,95%CI（0.63-2.74） |
| Shushan A(b)(*5*) | 1996 | Israel | case control research | BOT | 1990-1993 | N_OI-OT_=10；N_OI-NOR_=29  N_CT-OT_=26；N_CT-NOR_=379 | OR=3.52，95%CI（1.23-10.09） |
| Berit Jul Mosgaard(*6*) | 1997 | Danish | case control research | IOC | 1989-1994 | N_OI-OT_=28；N_OI-NOR_=58  N_CT-OT_=585；N_CT-NOR_=1526 | Not directly described |
| Fabio Parazzini(*7*) | 1997 | Italy | case control research | IOC | 1983-1991 | N_OI-OT_=5；N_OI-NOR_=1  N_CT-OT_=966；N_CT-NOR_=2692 | OR=1.1,95%CI（0.4-3.3） |
| Berit Jul Mosgaard(*8*) | 1998 | Danish | case control research | BOT | 1989-1994 | N_OI-OT_=17；N_OI-NOR_=58  N_CT-OT_=198；N_CT-NOR_=1526 | OR=2.19,95%CI (1.24–3.85) |
| Baruch Modan(*9*) | 1998 | Israel | cohort study | IOC | 1987-1991 | N_OI-OT_=6；N_OI-NOR_=1265  N_CT-OT_=6；N_CT-NOR_=1139 | SIR=1.6,95%CI（0.8-2.9） |
| Potashnik G(*10*) | 1999 | Israel | cohort study | IOC | 1960-1984  （17.9Y） | N_OI-OT_=1；N_OI-NOR_=755  N_CT-OT_=1；N_CT-NOR_=404 | SIR=0.91,95%CI（0.1-3.27） |
| Alison Venn(*11*) | 1999 | Australia | cohort study | IOC | 1986-1994 | N_OI-OT_=7；N_OI-NOR_=20557  N_CT-OT_=6；N_CT-NOR_=8975 | SIR=0.88,95%CI（0.42-1.84） |
| Fabio Parazzini(*12*) | 2001 | Italy | case control research | IOC | 1992-1999 | N_OI-OT_=15；N_OI-NOR_=26  N_CT-OT_=1016；N_CT-NOR_=2385 | OR=1.7,95%CI（0.7-2.5） |
| Pat Doyle(*13*) | 2002 | England | cohort study | IOC | 1990-1997 | N_OI-OT_=4；N_OI-NOR_=4097  N_CT-OT_=2；N_CT-NOR_=1206 | OR=0.59,95%CI（0.12-3.00） |
| Roberta B. Ness`(*14*) | 2002 | America | case control research | IOC | 1989-1999 | N_OI-OT_=149；N_OI-NOR_=200  N_CT-OT_=911；N_CT-NOR_=1137 | OR=0.97,95%CI（0.76-1.25） |
| Louise A. Brinton(*15*) | 2004 | America | cohort study | IOC | 1978-1999 | N_OI-OT_=16；N_OI-NOR_=4127  N_CT-OT_=29；N_CT-NOR_=4226 | Not directly described |
| Mary Anne Rossing(*16*) | 2004 | America | case control research | IOC | 1994-1998 | N_OI-OT_=19；N_OI-NOR_=95  N_CT-OT_=358；N_CT-NOR_=1538 | Not directly described |
| Maite Cusido(*17*) | 2007 | Spain | case control research | BOT | 1982-2000 | N_OI-OT_=5；N_OI-NOR_=34  N_CT-OT_=37；N_CT-NOR_=223 | Not directly described |
| P.Kristiansson(a)(*18*) | 2007 | Sweden | cohort study | IOC | 1981-2001 | N_OI-OT_=3；N_OI-NOR_=8598  N_CT-OT_=314；N_CT-NOR_= 621173 | Not directly described |
| P.Kristiansson(b)(*18*) | 2007 | Sweden | cohort study | BOT | 1981-2001 | N_OI-OT_=8；N_OI-NOR_=8598  N_CT-OT_=245；N_CT-NOR_= 621173 | Not directly described |
| R. Calderon-Margalit(*19*) | 2008 | Israel | cohort study | IOC | 1974-2004 | N_OI-OT_=1；N_OI-NOR_=500  N_CT-OT_=42；N_CT-NOR_= 13315 | HR=0.61,95%CI（0.08-4.42） |
| Allan Jensen(*20*) | 2009 | Danish | cohort study | IOC | 1998-2006 | N_OI-OT_=77；N_OI-NOR_=615  N_CT-OT_=79；N_CT-NOR_= 626 | OR=1.03,95%CI（0.73-1.47） |
| [Dos](https://pubmed.ilibs.cn/?size=100&term=Silva+Idos+S&cauthor_id=19436296) Santos Silva(*21*) | 2009 | England | cohort study | IOC | 1971-2005 | N_OI-OT_=12；N_OI-NOR_=3168  N_CT-OT_=8；N_CT-NOR_= 3941 | SIR=1.10,95%CI（0.57-1.93） |
| Karin Sanner(a)(*22*) | 2009 | Sweden | cohort study | IOC | 1958-2004 | N_OI-OT_=9；N_OI-NOR_=1137  N_CT-OT_=8；N_CT-NOR_= 1602 | SIR=1.19,95%CI（0.54-2.25） |
| Karin Sanner(b)(*22*) | 2009 | Sweden | cohort study | BOT | 1958-2004 | N_OI-OT_=7；N_OI-NOR_=1137  N_CT-OT_=5；N_CT-NOR_= 1602 | SIR=3.61,95%CI（1.45-7.44） |
| F.E. van Leeuwen(a)(*23*) | 2011 | Netherlands | cohort study | IOC | 1989-2007 | N_OI-OT_=30；N_OI-NOR_=19085  N_CT-OT_=12；N_CT-NOR_= 5990 | HR=1.69,95%CI（0.54–2.41） |
| F.E. van Leeuwen(b)(*23*) | 2011 | Netherlands | cohort study | BOT | 1989-2007 | N_OI-OT_=31；N_OI-NOR_=19085  N_CT-OT_=4；N_CT-NOR_= 5990 | HR=6.38,95%CI（2.05–19.84） |
| Michelle L. Kurta(*24*) | 2012 | America | case control research | IOC | 2003-2008 | N_OI-OT_=50；N_OI-NOR_=102  N_CT-OT_=852；N_CT-NOR_= 1700 | OR=0.87,95%CI (0.54–1.40) |
| Lerner-Geva Liat(*25*) | 2012 | Israel | cohort study | IOC | 1964-2011 | N_OI-OT_=9；N_OI-NOR_=1178  N_CT-OT_=9；N_CT-NOR_= 1045 | Not directly described |
| Louise A. Brinton(*26*) | 2013 | Israel | cohort study | IOC | 1994-2011 | N_OI-OT_=34；N_OI-NOR_=66928  N_CT-OT_=11；N_CT-NOR_= 19524 | HR= 0.90,95%CI (0.45–1.79) |
| Britton Trabert(*27*) | 2013 | America | cohort study | IOC | 1988-2010 | N_OI-OT_=38；N_OI-NOR_=3707  N_CT-OT_=47；N_CT-NOR_= 6033 | Not directly described |
| Albert Asante(a)(*28*) | 2013 | America | case control research | IOC | 1999-2012 | N_OI-OT_=28；N_OI-NOR_=44  N_CT-OT_=160；N_CT-NOR_= 138 | Not directly described |
| Albert Asante(b)(*28*) | 2013 | America | case control research | BOT | 1999-2012 | N_OI-OT_=4；N_OI-NOR_=44  N_CT-OT_=34；N_CT-NOR_= 138 | Not directly described |
| S.M. Bjornholt (*29*) | 2015 | Danish | cohort study | BOT | 1963-2006 | N_OI-OT_=89；N_OI-NOR_=683  N_CT-OT_=53；N_CT-NOR_= 645 | RR=1.00,95%CI (0.67–1.51) |
| Jacek Gronwald(*30*) | 2015 | Canada | case control research | IOC | No description | N_OI-OT_=18；N_OI-NOR_=923  N_CT-OT_=27；N_CT-NOR_= 914 | Not directly described |
| R. Kessous(*31*) | 2016 | Israel | cohort study | IOC | 1988-2013 | N_OI-OT_=7；N_OI-NOR_=4356  N_CT-OT_=51；N_CT-NOR_=101617 | HR=3.9, 95 % CI (1.2–12.6) |
| Reigstad MM(a)(*32*) | 2017 | Norway | cohort study | IOC | 1960-2014 | N_OI-OT_=22；N_OI-NOR_=56156  N_CT-OT_=609；N_CT-NOR_=1297298 | HR=1.93,95%CI (1.18-3.16) |
| Reigstad MM(b)(*32*) | 2017 | Norway | cohort study | BOT | 1960-2014 | N_OI-OT_=16；N_OI-NOR_=56156  N_CT-OT_=623；N_CT-NOR_=1297298 | HR=0.97,95%CI (0.56-1.70) |
| Frida E. Lundberg(a)(*33*) | 2019 | Sweden | cohort study | IOC | 1982-2013 | N_OI-OT_=39；N_OI-NOR_=37959  N_CT-OT_=950；N_CT-NOR_=1300402 | HR=2.43,95%CI(1.73–3.42) |
| Frida E. Lundberg(b)(*33*) | 2019 | Sweden | cohort study | BOT | 1982-2013 | N_OI-OT_=27；N_OI-NOR_=37959  N_CT-OT_=720；N_CT-NOR_=1300402 | HR=1.91,95%CI (1.27–2.86) |
| Mandy Spaan(a)(*34*) | 2021 | Netherland | cohort study | IOC | 1983-2018 | N_OI-OT_=25；N_OI-NOR_=6477  N_CT-OT_=33；N_CT-NOR_=9651 | HR=0.93,95%CI (0.55-1.57) |
| Mandy Spaan(b)(*34*) | 2021 | Netherland | cohort study | BOT | 1983-2018 | N_OI-OT_=13；N_OI-NOR_=6477  N_CT-OT_=28；N_CT-NOR_=9651 | HR=0.62,95%CI (0.32- 1.20) |

IOC: invasive ovarian cancer

BOT: borderline ovarian tumor

OI-OT: ovarian tumor patients in ovulation induction group

OI-NOR: normal women in ovulation induction group

CT-OT: ovarian tumor patients in control group

CT-NOR: normal women in control group

HR: Hazard Ratio

RR: relative risk

OR: odds ratio

SIR: standardised incidence ratio

95%CI: 95% confidence intervals

1. R. Harris, A. S. Whittemore, J. Itnyre, Characteristics relating to ovarian cancer risk: collaborative analysis of 12 US case-control studies. III. Epithelial tumors of low malignant potential in white women. Collaborative Ovarian Cancer Group. *American journal of epidemiology* **136**, 1204-1211 (1992).

2. A. S. Whittemore, R. Harris, J. Itnyre, Characteristics relating to ovarian cancer risk: collaborative analysis of 12 US case-control studies. II. Invasive epithelial ovarian cancers in white women. Collaborative Ovarian Cancer Group. *American journal of epidemiology* **136**, 1184-1203 (1992).

3. S. Franceschi *et al.*, Fertility drugs and risk of epithelial ovarian cancer in Italy. *Human reproduction (Oxford, England)* **9**, 1673-1675 (1994).

4. A. Venn *et al.*, Breast and ovarian cancer incidence after infertility and in vitro fertilisation. *Lancet (London, England)* **346**, 995-1000 (1995).

5. A. Shushan *et al.*, Human menopausal gonadotropin and the risk of epithelial ovarian cancer. *Fertility and sterility* **65**, 13-18 (1996).

6. B. J. Mosgaard, O. Lidegaard, S. K. Kjaer, G. Schou, A. N. Andersen, Infertility, fertility drugs, and invasive ovarian cancer: a case-control study. *Fertility and sterility* **67**, 1005-1012 (1997).

7. F. Parazzini *et al.*, Treatment for infertility and risk of invasive epithelial ovarian cancer. *Human reproduction (Oxford, England)* **12**, 2159-2161 (1997).

8. B. J. Mosgaard, O. Lidegaard, S. K. Kjaer, G. Schou, A. N. Andersen, Ovarian stimulation and borderline ovarian tumors: a case-control study. *Fertility and sterility* **70**, 1049-1055 (1998).

9. B. Modan *et al.*, Cancer incidence in a cohort of infertile women. *American journal of epidemiology* **147**, 1038-1042 (1998).

10. G. Potashnik *et al.*, Fertility drugs and the risk of breast and ovarian cancers: results of a long-term follow-up study. *Fertility and sterility* **71**, 853-859 (1999).

11. A. Venn, L. Watson, F. Bruinsma, G. Giles, D. Healy, Risk of cancer after use of fertility drugs with in-vitro fertilisation. *Lancet (London, England)* **354**, 1586-1590 (1999).

12. F. Parazzini *et al.*, Use of fertility drugs and risk of ovarian cancer. *Human reproduction (Oxford, England)* **16**, 1372-1375 (2001).

13. P. Doyle, N. Maconochie, V. Beral, A. J. Swerdlow, S. L. Tan, Cancer incidence following treatment for infertility at a clinic in the UK. *Human reproduction (Oxford, England)* **17**, 2209-2213 (2002).

14. R. B. Ness *et al.*, Infertility, fertility drugs, and ovarian cancer: a pooled analysis of case-control studies. *American journal of epidemiology* **155**, 217-224 (2002).

15. L. A. Brinton *et al.*, Ovarian cancer risk after the use of ovulation-stimulating drugs. *Obstetrics and gynecology* **103**, 1194-1203 (2004).

16. M. A. Rossing, M. T. Tang, E. W. Flagg, L. K. Weiss, K. G. Wicklund, A case-control study of ovarian cancer in relation to infertility and the use of ovulation-inducing drugs. *American journal of epidemiology* **160**, 1070-1078 (2004).

17. M. Cusidó, R. Fábregas, B. S. Pere, C. Escayola, P. N. Barri, Ovulation induction treatment and risk of borderline ovarian tumors. *Gynecological endocrinology : the official journal of the International Society of Gynecological Endocrinology* **23**, 373-376 (2007).

18. P. Kristiansson, O. Björ, H. Wramsby, Tumour incidence in Swedish women who gave birth following IVF treatment. *Human reproduction (Oxford, England)* **22**, 421-426 (2007).

19. R. Calderon-Margalit *et al.*, Cancer risk after exposure to treatments for ovulation induction. *American journal of epidemiology* **169**, 365-375 (2009).

20. A. Jensen, H. Sharif, K. Frederiksen, S. K. Kjaer, Use of fertility drugs and risk of ovarian cancer: Danish Population Based Cohort Study. *BMJ (Clinical research ed.)* **338**, b249 (2009).

21. S. Silva Idos *et al.*, Ovulation-stimulation drugs and cancer risks: a long-term follow-up of a British cohort. *British journal of cancer* **100**, 1824-1831 (2009).

22. K. Sanner *et al.*, Ovarian epithelial neoplasia after hormonal infertility treatment: long-term follow-up of a historical cohort in Sweden. *Fertility and sterility* **91**, 1152-1158 (2009).

23. F. E. van Leeuwen *et al.*, Risk of borderline and invasive ovarian tumours after ovarian stimulation for in vitro fertilization in a large Dutch cohort. *Human reproduction (Oxford, England)* **26**, 3456-3465 (2011).

24. M. L. Kurta *et al.*, Use of fertility drugs and risk of ovarian cancer: results from a U.S.-based case-control study. *Cancer epidemiology, biomarkers & prevention : a publication of the American Association for Cancer Research, cosponsored by the American Society of Preventive Oncology* **21**, 1282-1292 (2012).

25. L. Lerner-Geva *et al.*, Are infertility treatments a potential risk factor for cancer development? Perspective of 30 years of follow-up. *Gynecological endocrinology : the official journal of the International Society of Gynecological Endocrinology* **28**, 809-814 (2012).

26. L. A. Brinton *et al.*, In vitro fertilization and risk of breast and gynecologic cancers: a retrospective cohort study within the Israeli Maccabi Healthcare Services. *Fertility and sterility* **99**, 1189-1196 (2013).

27. B. Trabert *et al.*, Ovulation-inducing drugs and ovarian cancer risk: results from an extended follow-up of a large United States infertility cohort. *Fertility and sterility* **100**, 1660-1666 (2013).

28. A. Asante *et al.*, Fertility drug use and the risk of ovarian tumors in infertile women: a case-control study. *Fertility and sterility* **99**, 2031-2036 (2013).

29. S. M. Bjørnholt, S. K. Kjaer, T. S. Nielsen, A. Jensen, Risk for borderline ovarian tumours after exposure to fertility drugs: results of a population-based cohort study. *Human reproduction (Oxford, England)* **30**, 222-231 (2015).

30. J. Gronwald *et al.*, Treatment of infertility does not increase the risk of ovarian cancer among women with a BRCA1 or BRCA2 mutation. *Fertility and sterility* **105**, 781-785 (2016).

31. R. Kessous, E. Davidson, M. Meirovitz, R. Sergienko, E. Sheiner, The risk of female malignancies after fertility treatments: a cohort study with 25-year follow-up. *Journal of cancer research and clinical oncology* **142**, 287-293 (2016).

32. M. M. Reigstad *et al.*, Cancer Risk in Women Treated with Fertility Drugs According to Parity Status-A Registry-based Cohort Study. *Cancer epidemiology, biomarkers & prevention : a publication of the American Association for Cancer Research, cosponsored by the American Society of Preventive Oncology* **26**, 953-962 (2017).

33. F. E. Lundberg, A. L. V. Johansson, K. Rodriguez-Wallberg, K. Gemzell-Danielsson, A. N. Iliadou, Assisted reproductive technology and risk of ovarian cancer and borderline tumors in parous women: a population-based cohort study. *European journal of epidemiology* **34**, 1093-1101 (2019).

34. M. Spaan *et al.*, Long-Term Risk of Ovarian Cancer and Borderline Tumors After Assisted Reproductive Technology. *Journal of the National Cancer Institute* **113**, 699-709 (2021).
