# Supplementary material for: Ovulation induction drug and ovarian cancer: an updated systematic review and meta-analysis
Source: J Ovarian Res. 2023 Jan 24;16:22. doi: 10.1186/s13048-022-01084-z (PMC9872323; doi:10.1186/s13048-022-01084-z)
Supplement: Supplementary file 5 — Additional file 5: Supplementary Table S3a. Ovarian tumor in the nulliparous women. Supplementary Table S3b. Ovarian tumor in the multiparous women. [file 13048_2022_1084_MOESM5_ESM.docx]

Supplementary Table S3a: ovarian tumor in the nulliparous women.

| Author | Year | Study type | Tumor type | Women in nulligravid subgroups | Endpoint evaluation |
| --- | --- | --- | --- | --- | --- |
| Alice S. Whittemore | 1992 | case control research | IOC | N_OI-OT_=12；N_OI-NOR_=1  N_CT-OT_=76；N_CT-NOR_=100 | OR=27.0,95%CI（2.3-315.6） |
| Berit Jul Mosgaard | 1997 | case control research | IOC | N_OI-OT_=18；N_OI-NOR_=19  N_CT-OT_=66；N_CT-NOR_=92 | OR=2.26,95%CI（0.92-5.58） |
| Berit Jul Mosgaard | 1998 | case control research | BOT | N_OI-OT_=10；N_OI-NOR_=19  N_CT-OT_=27；N_CT-NOR_=92 | OR=2.88,95%CI（0.95-8.74） |
| Fabio Parazzini | 2001 | case control research | IOC | N_OI-OT_=3；N_OI-NOR_=11  N_CT-OT_=181；N_CT-NOR_=370 | OR=0.6,95%CI（0.1-2.0） |
| Roberta B. Ness | 2002 | case control research | IOC | N_OI-OT_=54；N_OI-NOR_=22  N_CT-OT_=191；N_CT-NOR_=147 | OR=0.60,95%CI（0.90-2.87） |
| Mary Anne Rossing | 2004 | case control research | IOC | N_OI-OT_=6；N_OI-NOR_=20  N_CT-OT_=102；N_CT-NOR_=322 | OR=1.0,95%CI（0.4-2.8） |
| Michelle L. Kurta | 2012 | case control research | IOC | N_OI-OT_=27；N_OI-NOR_=23  N_CT-OT_=186；N_CT-NOR_=207 | OR=1.52,95%CI（0.68-3.41） |
| Louise A. Brinton | 2013 | cohort study | IOC | N_OI-OT_=19；N_OI-NOR_=17379  N_CT-OT_=8；N_CT-NOR_=5690 | HR=0.71,95%CI（0.31-1.64） |
| Sarah Marie Bjornholt | 2014 | cohort study | BOT | N_OI-OT_=50；N_OI-NOR_=227  N_CT-OT_=32；N_CT-NOR_=192 | RR=0.95,95%CI（0.57-1.58） |
| Reigstad MM(a) | 2017 | cohort study | IOC | N_OI-OT_=14；N_OI-NOR_=9483  N_CT-OT_=219；N_CT-NOR_= 579201 | HR=2.49,95%CI（1.30-4.78） |
| Reigstad MM(b) | 2017 | cohort study | BOT | N_OI-OT_=7；N_OI-NOR_=9483  N_CT-OT_=246；N_CT-NOR_= 579201 | HR=1.16,95%CI（0.49-2.73） |

Supplementary Table S3b: ovarian tumor in the multiparous women.

| Author | Year | Study type | Tumor type | Women in gravid subgroups | Endpoint evaluation |
| --- | --- | --- | --- | --- | --- |
| Alice S. Whittemore | 1992 | case control research | IOC | N_OI-OT_=8；N_OI-NOR_=10  N_CT-OT_=526；N_CT-NOR_=990 | OR=1.4,95%CI（0.52-3.6） |
| Silvia Franceschi | 1994 | case control research | IOC | N_OI-OT_=2；N_OI-NOR_=10  N_CT-OT_=157；N_CT-NOR_=1152 | OR=1.35,95%CI（0.29-6.32） |
| Berit Jul Mosgaard | 1997 | case control research | IOC | N_OI-OT_=10；N_OI-NOR_=39  N_CT-OT_=519；N_CT-NOR_=1434 | OR=0.73,95%CI（0.29-1.82） |
| Berit Jul Mosgaard | 1998 | case control research | BOT | N_OI-OT_=7；N_OI-NOR_=39  N_CT-OT_=171；N_CT-NOR_=1434 | OR=1.11,95%CI（0.37-3.30） |
| Fabio Parazzini | 2001 | case control research | IOC | N_OI-OT_=12；N_OI-NOR_=15  N_CT-OT_=835；N_CT-NOR_=2015 | OR=1.9,95%CI（0.9-4.1） |
| Roberta B. Ness | 2002 | case control research | IOC | N_OI-OT_=95；N_OI-NOR_=178  N_CT-OT_=720；N_CT-NOR_=990 | OR=0.82,95%CI（0.62-1.09） |
| Mary Anne Rossing | 2004 | case control research | IOC | N_OI-OT_=13；N_OI-NOR_=75  N_CT-OT_=256；N_CT-NOR_=1216 | OR=0.8,95%CI（0.4-1.5） |
| R. Calderon-Margalit | 2008 | cohort study | IOC | N_OI-OT_=1；N_OI-NOR_=308  N_CT-OT_=15；N_CT-NOR_=5108 | HR=0.88,95%CI（0.12-6.74） |
| Michelle L. Kurta | 2012 | case control research | IOC | N_OI-OT_=23；N_OI-NOR_=79  N_CT-OT_=666；N_CT-NOR_=1493 | OR=0.72,95%CI（0.94-1.19） |
| Louise A. Brinton | 2013 | cohort study | IOC | N_OI-OT_=15；N_OI-NOR_=49772  N_CT-OT_=3；N_CT-NOR_=13954 | HR=1.32,95%CI（0.38-4.59） |
| Sarah Marie Bjornholt | 2014 | cohort study | BOT | N_OI-OT_=39；N_OI-NOR_=456  N_CT-OT_=21；N_CT-NOR_=453 | RR=1.07,95%CI（0.59-1.95） |
| Reigstad MM(a) | 2017 | cohort study | IOC | N_OI-OT_=8；N_OI-NOR_=28980  N_CT-OT_=390；N_CT-NOR_= 698665 | HR=1.37,95%CI（0.64-2.96） |
| Reigstad MM(b) | 2017 | cohort study | BOT | N_OI-OT_=9；N_OI-NOR_=28980  N_CT-OT_=377；N_CT-NOR_= 698665 | HR=0.87,95%CI（0.41-1.82） |

IOC: invasive ovarian cancer

BOT: borderline ovarian tumor

OI-OT: ovarian tumor patients in ovulation induction group

OI-NOR: normal women in ovulation induction group

CT-OT: ovarian tumor patients in control group

CT-NOR: normal women in control group

HR: Hazard Ratio

RR: relative risk

OR: odds ratio

95%CI: 95% confidence intervals
