# Supplementary material for: Ovulation induction drug and ovarian cancer: an updated systematic review and meta-analysis
Source: J Ovarian Res. 2023 Jan 24;16:22. doi: 10.1186/s13048-022-01084-z (PMC9872323; doi:10.1186/s13048-022-01084-z)
Supplement: Supplementary file 6 — Additional file 6: Supplementary Table S4. Ovarian tumor between the nulliparous and multiparous group. [file 13048_2022_1084_MOESM6_ESM.docx]

Supplementary Table S4: ovarian tumor between the nulliparous and multiparous group.

| Author | Year | Study type | Tumor type | Women in subgroups | Endpoint evaluation |
| --- | --- | --- | --- | --- | --- |
| Alice S. Whittemore | 1992 | case control research | IOC | N_nulli-OT_=12；N _nulli-NOR_=1  N_multi-OT_=8；N_multi-NOR_=10 | Not directly described |
| Berit Jul Mosgaard | 1997 | case control research | IOC | N_nulli-OT_=18；N _nulli-NOR_=19  N_multi-OT_=10；N_multi-NOR_=39 | Not directly described |
| Berit Jul Mosgaard | 1998 | case control research | BOT | N_nulli-OT_=10；N _nulli-NOR_=19  N_multi-OT_=7；N_multi-NOR_=39 | Not directly described |
| Fabio Parazzini | 2001 | case control research | IOC | N_nulli-OT_=3；N _nulli-NOR_=11  N_multi-OT_=12；N_multi-NOR_=15 | Not directly described |
| Roberta B. Ness | 2002 | case control research | IOC | N_nulli-OT_=54；N _nulli-NOR_=22  N_multi-OT_=95；N_multi-NOR_=178 | Not directly described |
| Mary Anne Rossing | 2004 | case control research | IOC | N_nulli-OT_=6；N _nulli-NOR_=20  N_multi-OT_=13；N_multi-NOR_=75 | Not directly described |
| Michelle L. Kurta | 2012 | case control research | IOC | N_nulli-OT_=27；N _nulli-NOR_=23  N_multi-OT_=23；N_multi-NOR_=79 | Not directly described |
| Louise A. Brinton | 2013 | cohort study | IOC | N_nulli-OT_=19；N _nulli-NOR_=17379  N_multi-OT_=15；N_multi-NOR_=49772 | Not directly described |
| Sarah Marie Bjornholt | 2014 | cohort study | BOT | N_nulli-OT_=50；N _nulli-NOR_=227  N_multi-OT_=39；N_multi-NOR_=456 | Not directly described |
| Reigstad MM(a) | 2017 | cohort study | IOC | N_nulli-OT_=14；N _nulli-NOR_=9483  N_multi-OT_=8；N_multi-NOR_=28980 | Not directly described |
| Reigstad MM(b) | 2017 | cohort study | BOT | N_nulli-OT_=7；N _nulli-NOR_=9483  N_multi-OT_=9；N_multi-NOR_=28980 | Not directly described |

IOC: invasive ovarian cancer

BOT: borderline ovarian tumor

nulli-OT: ovarian tumor patients in nulliparous group who were on ovulation induction therapy

nulli-NOR: normal women in nulliparous n group who were on ovulation induction therapy

multi-OT: ovarian tumor patients in multiparous group who were on ovulation induction therapy

multi-NOR: normal women in multiparous group who were on ovulation induction therapy
