# Supplementary material for: Ovulation induction drug and ovarian cancer: an updated systematic review and meta-analysis
Source: J Ovarian Res. 2023 Jan 24;16:22. doi: 10.1186/s13048-022-01084-z (PMC9872323; doi:10.1186/s13048-022-01084-z)
Supplement: Supplementary file 7 — Additional file 7: Supplementary Table S5a. Ovarian tumor and stimulation cycles(< 3 cycles). Supplementary Table S5b. Ovarian tumor and stimulation cycles(≥3 cycles). Supplementary Table S5c. Ovarian tumor and stimulation cycles(< 6 cycles). Supplementary Table S5d. Ovarian tumor and stimulation cycles(≥6 cycles). Supplementary Table S5e. Ovarian tumor and stimulation cycles(< 12 cycles). Supplementary Table S5f. Ovarian tumor and stimulation cycles(≥12 cycles). [file 13048_2022_1084_MOESM7_ESM.docx]

Supplementary Table S5a: ovarian tumor and stimulation cycles（<3 cycles）.

| Author | Year | Study type | Tumor type | Women in subgroups | Endpoint evaluation |
| --- | --- | --- | --- | --- | --- |
| Roberta B. Ness | 2002 | case control research | IOC | N_OT<3 cycles_=48；N_NOR<3 cycles_=72  N_CT-OT_=911；N_CT-NOR_=1137 | Not directly described |
| Karin Sanner | 2008 | cohort study | IOC | N_OT<3 cycles_=7；N_NOR<3 cycles_=336  N_CT-OT_=13；N_CT-NOR_=1602 | Not directly described |
| F.E. van Leeuwen | 2011 | cohort study | IOC | N_OT<3 cycles_=11；N_NOR<3 cycles_=6282  N_CT-OT_=12；N_CT-NOR_=5990 | SIR=1.35,95%CI（0.68-2.42） |
| Mandy Spaan(a) | 2020 | cohort study | IOC | N_OT<3 cycles_=50；N_NOR<3 cycles_=12405  N_CT-OT_=37；N_CT-NOR_=9856 | HR=1.18,95%CI（0.76-1.82） |

Supplementary Table S5b: ovarian tumor and stimulation cycles（≥3 cycles）.

| Author | Year | Study type | Tumor type | Women in subgroups | Endpoint evaluation |
| --- | --- | --- | --- | --- | --- |
| Roberta B. Ness | 2002 | case control research | IOC | N_OT≥3 cycles_=101；N_NOR≥3 cycles_=128  N_CT-OT_=911；N_CT-NOR_=1137 | Not directly described |
| Karin Sanner | 2008 | cohort study | IOC | N_OT≥3 cycles_=4；N_NOR≥3 cycles_=307  N_CT-OT_=13；N_CT-NOR_=1602 | Not directly described |
| F.E. van Leeuwen | 2011 | cohort study | IOC | N_OT≥3 cycles_=17；N_NOR≥3 cycles_=9589  N_CT-OT_=12；N_CT-NOR_=5990 | Not directly described |
| Mandy Spaan(a) | 2020 | cohort study | IOC | N_OT≥3 cycles_=65；N_NOR≥3 cycles_=17977  N_CT-OT_=37；N_CT-NOR_=9856 | Not directly described |

Supplementary Table S5c: ovarian tumor and stimulation cycles（<6 cycles）.

| Author | Year | Study type | Tumor type | Women in subgroups | Endpoint evaluation |
| --- | --- | --- | --- | --- | --- |
| Fabio Parazzini | 1997 | case control research | IOC | N_OT<6 cycles_=1；N_NOR<6 cycles_=3  N_CT-OT_=966；N_CT-NOR_=2692 | OR=0.7,95%CI（0.1-7.9） |
| Alison Venn | 1999 | cohort study | IOC | N_OT<6 cycles_=4；N_NOR<6 cycles_=15143  N_CT-OT_=6；N_CT-NOR_=8975 | Not directly described |
| Potashnik G | 1999 | cohort study | IOC | N_OT<6 cycles_=1；N_NOR<6 cycles_=506  N_CT-OT_=1；N_CT-NOR_=404 | Not directly described |
| Mary Anne Rossing | 2004 | case control research | IOC | N_OT<6 cycles_=9；N_NOR<6 cycles_=41  N_CT-OT_=358；N_CT-NOR_=1538 | Not directly described |
| Michelle L. Kurta | 2012 | case control research | IOC | N_OT<6 cycles_=22；N_NOR<6 cycles_=41  N_CT-OT_=105；N_CT-NOR_=192 | OR=0.92,95%CI（0.48-1.74） |

Supplementary Table S5d: ovarian tumor and stimulation cycles（≥6 cycles）.

| Author | Year | Study type | Tumor type | Women in subgroups | Endpoint evaluation |
| --- | --- | --- | --- | --- | --- |
| Fabio Parazzini | 1997 | case control research | IOC | N_OT≥6 cycles_=4；N_NOR≥6 cycles_=7  N_CT-OT_=966；N_CT-NOR_=2692 | OR=1.0,95%CI（0.2-3.8） |
| Alison Venn | 1999 | cohort study | IOC | N_OT≥6 cycles_=1；N_NOR≥6 cycles_=1924  N_CT-OT_=6；N_CT-NOR_=8975 | Not directly described |
| Mary Anne Rossing | 2004 | case control research | IOC | N_OT≥6 cycles_=7；N_NOR≥6 cycles_=42  N_CT-OT_=358；N_CT-NOR_=1538 | Not directly described |
| Michelle L. Kurta | 2012 | case control research | IOC | N_OT≥6 cycles_=27；N_NOR≥6 cycles_=57  N_CT-OT_=105；N_CT-NOR_=192 | OR=0.75,95%CI（0.42-1.34） |

Supplementary Table S5e: ovarian tumor and stimulation cycles（<12 cycles）.

| Author | Year | Study type | Tumor type | Women in subgroups | Endpoint evaluation |
| --- | --- | --- | --- | --- | --- |
| Roberta B. Ness | 2002 | case control research | IOC | N_OT<12 cycles_=119；N_NOR<12 cycles_=156  N_CT-OT_=991；N_CT-NOR_=1137 | Not directly described |
| Mary Anne Rossing | 2004 | case-control research | IOC | N_OT<12 cycles_=14；N_NOR<12 cycles_=71  N_CT-OT_=358；N_CT-NOR_=1538 | Not directly described |

Supplementary Table S5f: ovarian tumor and stimulation cycles（≥12 cycles）.

| Author | Year | Study type | Tumor type | Women in subgroups | Endpoint evaluation |
| --- | --- | --- | --- | --- | --- |
| Roberta B. Ness | 2002 | case control research | IOC | N_OT≥12 cycles_=30；N_NOR≥12 cycles_=44  N_CT-OT_=991；N_CT-NOR_=1137 | Not directly described |
| Mary Anne Rossing | 2004 | case control research | IOC | N_OT≥12 cycles_=2；N_NOR≥12 cycles_=12  N_CT-OT_=358；N_CT-NOR_=1538 | Not directly described |

IOC: invasive ovarian cancer

OT: ovarian tumor patients in ovulation induction group

NOR: normal women in ovulation induction group

CT-OT: ovarian tumor patients in control group

CT-NOR: normal women in control group

HR: Hazard Ratio

OR: odds ratio

SIR: standardised incidence ratio

95%CI: 95% confidence intervals
