# Supplementary material for: Ovulation induction drug and ovarian cancer: an updated systematic review and meta-analysis
Source: J Ovarian Res. 2023 Jan 24;16:22. doi: 10.1186/s13048-022-01084-z (PMC9872323; doi:10.1186/s13048-022-01084-z)
Supplement: Supplementary file 8 — Additional file 8: Supplementary Table S6a. Ovarian tumor in women who ever received CC. Supplementary Table S6b. Ovarian tumor in women who ever received HMG. Supplementary Table S6c. Ovarian tumor in women who ever received HCG. Supplementary Table S6d. Ovarian tumor in women who ever received GDT. Supplementary Table S6e. Ovarian tumor in women who ever received GnRH-a. [file 13048_2022_1084_MOESM8_ESM.docx]

Supplementary Table S6a: ovarian tumor in women who ever received CC.

| Author | Year | Study type | Tumor type | Women in subgroups | Endpoint evaluation |
| --- | --- | --- | --- | --- | --- |
| Asher Shushan(a) | 1996 | case control research | IOC | N_OI-OT_=11；N_OI-NOR_=18  N_CT-OT_=140；N_CT-NOR_=379 | OR=0.88,95%CI（0.33-2.34） |
| Asher Shushan(b) | 1996 | case control research | BOT | N_OI-OT_=2；N_OI-NOR_=18  N_CT-OT_=26；N_CT-NOR_=379 | OR=1.28,95%CI（0.25-6.87） |
| Berit Jul Mosgaard | 1997 | case control research | IOC | N_OI-OT_=23；N_OI-NOR_=40  N_CT-OT_=585；N_CT-NOR_=1526 | Not directly described |
| Berit Jul Mosgaard | 1998 | case control research | BOT | N_OI-OT_=15；N_OI-NOR_=40  N_CT-OT_=198；N_CT-NOR_=1526 | Not directly described |
| Baruch Modan | 1998 | cohort study | IOC | N_OI-OT_=6；N_OI-NOR_=1108  N_CT-OT_=6；N_CT-NOR_=1139 | Not directly described |
| Alison Venn | 1999 | cohort study | IOC | N_OI-OT_=3；N_OI-NOR_=7722  N_CT-OT_=6；N_CT-NOR_=8975 | Not directly described |
| Potashnik G | 1999 | cohort study | IOC | N_OI-OT_=1；N_OI-NOR_=755  N_CT-OT_=1；N_CT-NOR_=404 | Not directly described |
| Roberta B. Ness | 2002 | case control research | IOC | N_OI-OT_=42；N_OI-NOR_=64  N_CT-OT_=892；N_CT-NOR_=1070 | Not directly described |
| Louise A. Brinton | 2004 | cohort study | IOC | N_OI-OT_=15；N_OI-NOR_=3262  N_CT-OT_=29；N_CT-NOR_=4226 | Not directly described |
| Mary Anne Rossing | 2004 | cohort study | IOC | N_OI-OT_=16；N_OI-NOR_=82  N_CT-OT_=358；N_CT-NOR_=1538 | Not directly described |
| Cusido M | 2007 | case control research | BOT | N_OI-OT_=4；N_OI-NOR_=16  N_CT-OT_=37；N_CT-NOR_=223 | Not directly described |
| R. Calderon-Margalit | 2008 | cohort study | IOC | N_OI-OT_=1；N_OI-NOR_=310  N_CT-OT_=42；N_CT-NOR_=13315 | HR=0.98,95%CI（0.14-7.11） |
| Karin Sanner(a) | 2009 | cohort study | IOC | N_OI-OT_=3；N_OI-NOR_=822  N_CT-OT_=8；N_CT-NOR_=1602 | Not directly described |
| Karin Sanner(b) | 2009 | cohort study | BOT | N_OI-OT_=6；N_OI-NOR_=822  N_CT-OT_=5；N_CT-NOR_=1602 | Not directly described |
| Dos Santos Silva | 2009 | cohort study | IOC | N_OI-OT_=12；N_OI-NOR_=2972  N_CT-OT_=8；N_CT-NOR_=3941 | Not directly described |
| Allan Jensen | 2009 | cohort study | IOC | N_OI-OT_=58；N_OI-NOR_=417  N_CT-OT_=79；N_CT-NOR_=626 | RR=1.14,95%CI（0.79-1.64） |
| Michelle L. Kurta | 2012 | case control research | IOC | N_OI-OT_=37；N_OI-NOR_=72  N_CT-OT_=105；N_CT-NOR_=192 | Not directly described |
| Lerner-Geva Liat | 2012 | cohort study | IOC | N_OI-OT_=8；N_OI-NOR_=1034  N_CT-OT_=9；N_CT-NOR_=1045 | Not directly described |
| Britton Trabert | 2013 | cohort study | IOC | N_OI-OT_=37；N_OI-NOR_=3078  N_CT-OT_=47；N_CT-NOR_=6032 | RR=1.34,95%CI（0.86-2.07） |
| Sarah Marie Bjornholt | 2015 | cohort study | BOT | N_OI-OT_=56；N_OI-NOR_=440  N_CT-OT_=53；N_CT-NOR_=645 | RR=0.96,95%CI（0.64-1.44） |
| Reigstad MM(a) | 2017 | cohort study | IOC | N_OI-OT_=22；N_OI-NOR_= 56156  N_CT-OT_=609；N_CT-NOR_= 1297298 | HR=1.93,95%CI（1.18-3.16） |
| Reigstad MM(b) | 2017 | cohort study | BOT | N_OI-OT_=16；N_OI-NOR_= 56156  N_CT-OT_=623；N_CT-NOR_= 1297298 | HR=0.97,95%CI（0.56-1.70） |
| Mandy Spaan(a) | 2020 | cohort study | IOC | N_OI-OT_=25；N_OI-NOR_= 6477  N_CT-OT_=33；N_CT-NOR_= 9651 | HR=0.93,95%CI（0.55-1.57） |
| Mandy Spaan(b) | 2020 | cohort study | BOT | N_OI-OT_=13；N_OI-NOR_= 6477  N_CT-OT_=28；N_CT-NOR_= 9651 | HR=0.62,95%CI（0.32-1.20） |

Supplementary Table S6b: ovarian tumor in women who ever received HMG.

| Author | Year | Study type | Tumor type | Women in subgroups | Endpoint evaluation |
| --- | --- | --- | --- | --- | --- |
| Asher Shushan(a) | 1996 | case control research | IOC | N_OI-OT_=11；N_OI-NOR_=6  N_CT-OT_=140；N_CT-NOR_=379 | OR=3.19,95%CI（0.86-11.82） |
| Asher Shushan(b) | 1996 | case control research | BOT | N_OI-OT_=6；N_OI-NOR_=6  N_CT-OT_=26；N_CT-NOR_=379 | OR=9.38,95%CI（1.66-52.08） |
| Berit Jul Mosgaard | 1997 | case control research | IOC | N_OI-OT_=7；N_OI-NOR_=13  N_CT-OT_=107；N_CT-NOR_=187 | Not directly described |
| Berit Jul Mosgaard | 1998 | case control research | BOT | N_OI-OT_=5；N_OI-NOR_=13  N_CT-OT_=36；N_CT-NOR_=187 | Not directly described |
| Alison Venn | 1999 | cohort study | IOC | N_OI-OT_=4；N_OI-NOR_=19080  N_CT-OT_=6；N_CT-NOR_=8975 | Not directly described |
| Roberta B. Ness | 2002 | case control research | IOC | N_OI-OT_=19；N_OI-NOR_=24  N_CT-OT_=916；N_CT-NOR_=1110 | Not directly described |
| Lerner-Geva Liat | 2012 | cohort study | IOC | N_OI-OT_=1；N_OI-NOR_=369  N_CT-OT_=9；N_CT-NOR_=1045 | SIR=0.74,95%CI（0.01-4.12） |

Supplementary Table S6c: ovarian tumor in women who ever received HCG.

| Author | Year | Study type | Tumor type | Women in subgroups | Endpoint evaluation |
| --- | --- | --- | --- | --- | --- |
| Berit Jul Mosgaard | 1997 | case control research | IOC | N_OI-OT_=15；N_OI-NOR_=26  N_CT-OT_=107；N_CT-NOR_=187 | Not directly described |
| Berit Jul Mosgaard | 1998 | case control research | BOT | N_OI-OT_=13；N_OI-NOR_=26  N_CT-OT_=36；N_CT-NOR_=187 | Not directly described |
| Allan Jensen | 2009 | cohort study | IOC | N_OI-OT_=49；N_OI-NOR_=413  N_CT-OT_=79；N_CT-NOR_=626 | RR=0.89,95%CI（0.62-1.29） |
| Sarah Marie Bjornholt | 2015 | cohort study | BOT | N_OI-OT_=65；N_OI-NOR_=448  N_CT-OT_=53；N_CT-NOR_=645 | RR=0.91,95%CI（0.61-1.36） |

Supplementary Table S6d: ovarian tumor in women who ever received GDT.

| Author | Year | Study type | Tumor type | Women in subgroups | Endpoint evaluation |
| --- | --- | --- | --- | --- | --- |
| Louise A. Brinton | 2004 | cohort study | IOC | N_OI-OT_=5；N_OI-NOR_=861  N_CT-OT_=29；N_CT-NOR_=4226 | Not directly described |
| Cusido M | 2007 | case control research | BOT | N_OI-OT_=3；N_OI-NOR_=26  N_CT-OT_=37；N_CT-NOR_=223 | Not directly described |
| Karin Sanner(a) | 2009 | cohort study | IOC | N_OI-OT_=7；N_OI-NOR_=753  N_CT-OT_=8；N_CT-NOR_=1602 | Not directly described |
| Karin Sanner(b) | 2009 | cohort study | BOT | N_OI-OT_=4；N_OI-NOR_=753  N_CT-OT_=5；N_CT-NOR_=1602 | Not directly described |
| Dos Santos Silva | 2009 | cohort study | IOC | N_OI-OT_=5；N_OI-NOR_=1193  N_CT-OT_=8；N_CT-NOR_=3941 | Not directly described |
| Allan Jensen | 2009 | cohort study | IOC | N_OI-OT_=26；N_OI-NOR_=184  N_CT-OT_=79；N_CT-NOR_=626 | RR=0.83,95%CI（0.50-1.37） |
| Michelle L. Kurta | 2012 | case control research | IOC | N_OI-OT_=16；N_OI-NOR_=37  N_CT-OT_=105；N_CT-NOR_=192 | Not directly described |
| Britton Trabert | 2013 | cohort study | IOC | N_OI-OT_=8；N_OI-NOR_=944  N_CT-OT_=77；N_CT-NOR_=8803 | RR=1.00,95%CI（0.48-2.08） |
| Sarah Marie Bjornholt | 2015 | cohort study | BOT | N_OI-OT_=55；N_OI-NOR_=256  N_CT-OT_=53；N_CT-NOR_=645 | RR=1.32,95%CI（0.81-2.14） |

Supplementary Table S6e: ovarian tumor in women who ever received GnRH-a

| Author | Year | Study type | Tumor type | Women in subgroups | Endpoint evaluation |
| --- | --- | --- | --- | --- | --- |
| Alison Venn | 1999 | cohort study | IOC | N_OI-OT_=1；N_OI-NOR_=11152  N_CT-OT_=6；N_CT-NOR_=8975 | SIR=0·48,95%CI (0·07–3·38) |
| Allan Jensen | 2009 | cohort study | IOC | N_OI-OT_=15；N_OI-NOR_=110  N_CT-OT_=79；N_CT-NOR_=626 | RR=0.80,95%CI（0.51-1.42） |

IOC: invasive ovarian cancer

BOT: borderline ovarian tumor

OI-OT: ovarian tumor patients in ovulation induction group

OI-NOR: normal women in ovulation induction group

CT-OT: ovarian tumor patients in control group

CT-NOR: normal women in control group

HR: Hazard Ratio

RR: relative risk

OR: odds ratio

SIR: standardised incidence ratio

95%CI: 95% confidence intervals

CC: clomiphene citrate

HCG: human chorionic gonadotropin

HMG: human menopausal gonadotropin

GDT: gonadotrophin

GnRH-a: gonadotropin-releasing hormone analogues
