# Supplementary material for: Ovulation induction drug and ovarian cancer: an updated systematic review and meta-analysis
Source: J Ovarian Res. 2023 Jan 24;16:22. doi: 10.1186/s13048-022-01084-z (PMC9872323; doi:10.1186/s13048-022-01084-z)
Supplement: Supplementary file 9 — Additional file 9: Supplementary Table S7. Sensitivity analyses for the novel study during this update. [file 13048_2022_1084_MOESM9_ESM.docx]

| Outcome | OR | Lower 95% CI | Upper 95% CI |
| --- | --- | --- | --- |
| The risk of IOC between CC and CT group | 1.05 | 0.89 | 1.21 |
| The risk of BOT between CC and CT group | 1.73 | 0.96 | 2.50 |

Supplementary Table S7: Sensitivity analyses for the novel study during this update
